# Supplementary material for: Molecular elucidation of drug-induced abnormal assemblies of the hepatitis B virus capsid protein by solid-state NMR
Source: Nat Commun. 2023 Jan 28;14:471. doi: 10.1038/s41467-023-36219-3 (PMC9884277; doi:10.1038/s41467-023-36219-3)
Supplement: Supplementary file 1 — Supplementary Information [file 41467_2023_36219_MOESM1_ESM.pdf]

Molecular elucidation of drug-induced abnormal assemblies of the Hepatitis B Virus capsid protein by solid-state NMR

Lauriane Lecoq[1], Louis Brigandat[1], Rebecca Huber[1], Marie-Laure Fogeron[1], Shishan Wang[1], Marie Dujardin[1], Mathilde Briday[1], Thomas Wiegand[2,3,4], Morgane Callon[2], Alexander Malär[2], David Durantel[5], Dara Burdette[6], Jan Martin Berke[7], Beat H. Meier[2*], Michael Nassal[8*], Anja Böckmann[1*]

[1] Molecular Microbiology and Structural Biochemistry (MMSB), Labex Ecofect, UMR 5086 CNRS/Université de Lyon, 69367 Lyon, France

[2] Physical Chemistry, ETH Zurich, 8093 Zurich, Switzerland

[3] Current address: Max-Planck-Institute for Chemical Energy Conversion, Stiftstr. 34-36, 45470 Mülheim an der Ruhr, Germany

[4] Current address: Institute of Technical and Macromolecular Chemistry, RWTH Aachen University, Worringerweg 2, 52074 Aachen, Germany

[5] Centre de recherche en cancérologie de Lyon (CRCL), UMR 5286, Centre Léon Bérard, 69373 Lyon, France

[6] Gilead Sciences, Foster, CA, United States

[7] Janssen Pharmaceutica N.V., Beerse, Belgium

[8] Dept. of Medicine II / Molecular Biology, University of Freiburg

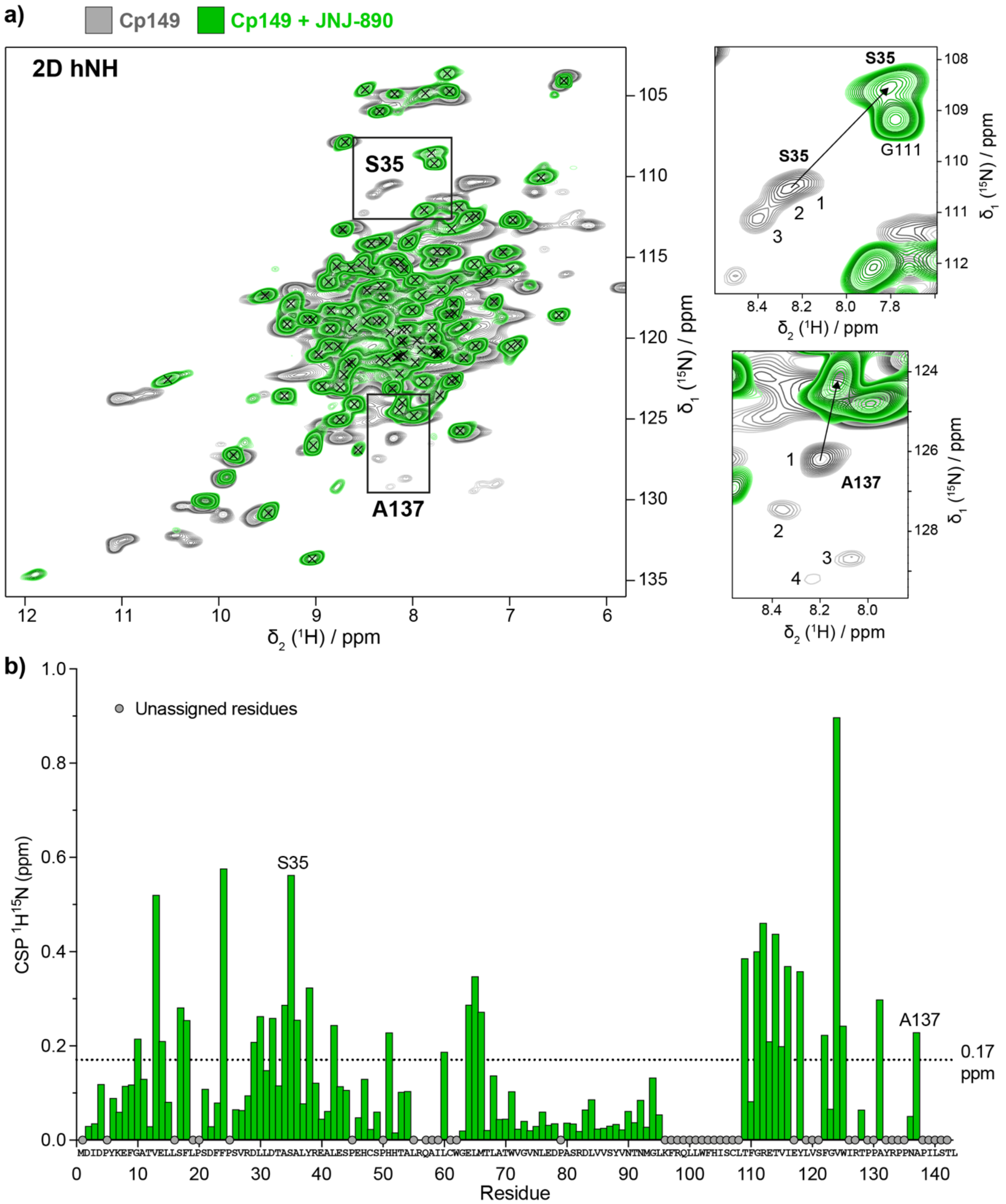

Supplementary Fig. 1: Effect of JNJ-890 on Cp149 capsids at the molecular level. a) Overlay of 2D hNH spectra of ^{2}H-^{13}C-^{15}N-Cp149 in absence (grey) and in presence of JNJ-890 (green, with cross-peaks corresponding to assigned residues) and extracts showing the A137 alanine and S35 regions. In both types of spectra, the peak splitting phenomenon due to subunits asymmetry disappears in presence of CAM-A. Spectra were recorded at 60 kHz MAS on a 800 MHz spectrometer. Corresponding EM micrographs are shown in Figure 1c. b) Chemical shift perturbations (CSPs) induced by the binding of JNJ-890 and opening of Cp149 capsids. The average CSP for all assigned residues is 0.17 ppm. Exact positions of peaks in the 2D hNH were determined using 3D hCANH spectra. For split peaks, the most intense signal was used for CSPs. n=1 independent experiments have been recorded. Source data are provided in the Source Data file.

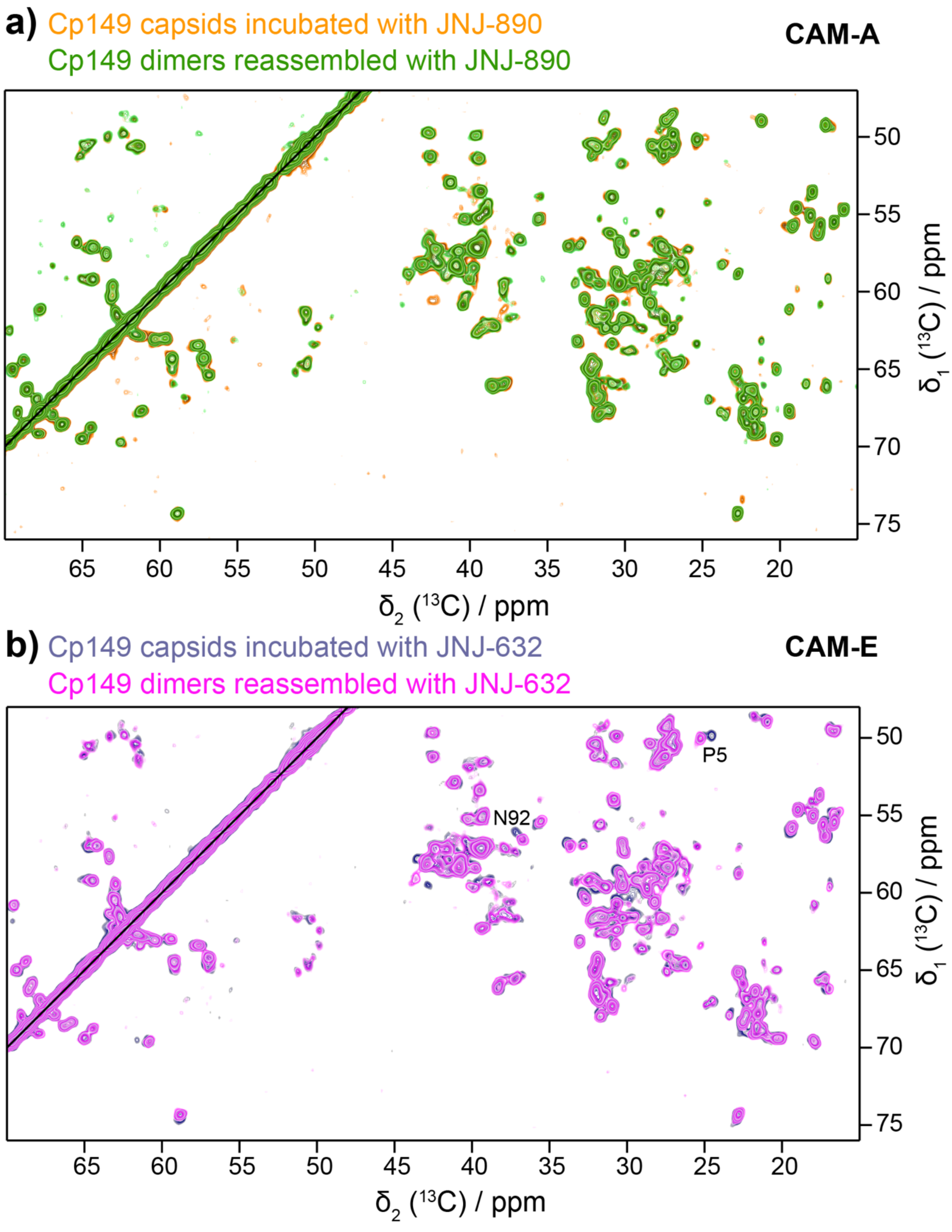

Supplementary Fig. 2: CAMs have a similar effect on Cp149 dimers and preformed Cp149 capsids. Zoom on aliphatic region of a 2D DARR of a) Cp149 capsid incubated with JNJ-890 for 2 hours at 37 °C (orange) and Cp149 dimer assembled with JNJ-890 overnight at room temperature (green); and b) Cp149 capsid incubated with JNJ-632 for 2 hours at 37 °C (grey) and Cp149 dimer reassembled with JNJ-632 overnight at room temperature (pink). The few differences observed in panel b) are due to the bound Triton X-100 in the hydrophobic pocket[1], which was not removed in this sample. n=1 independent experiments have been recorded.

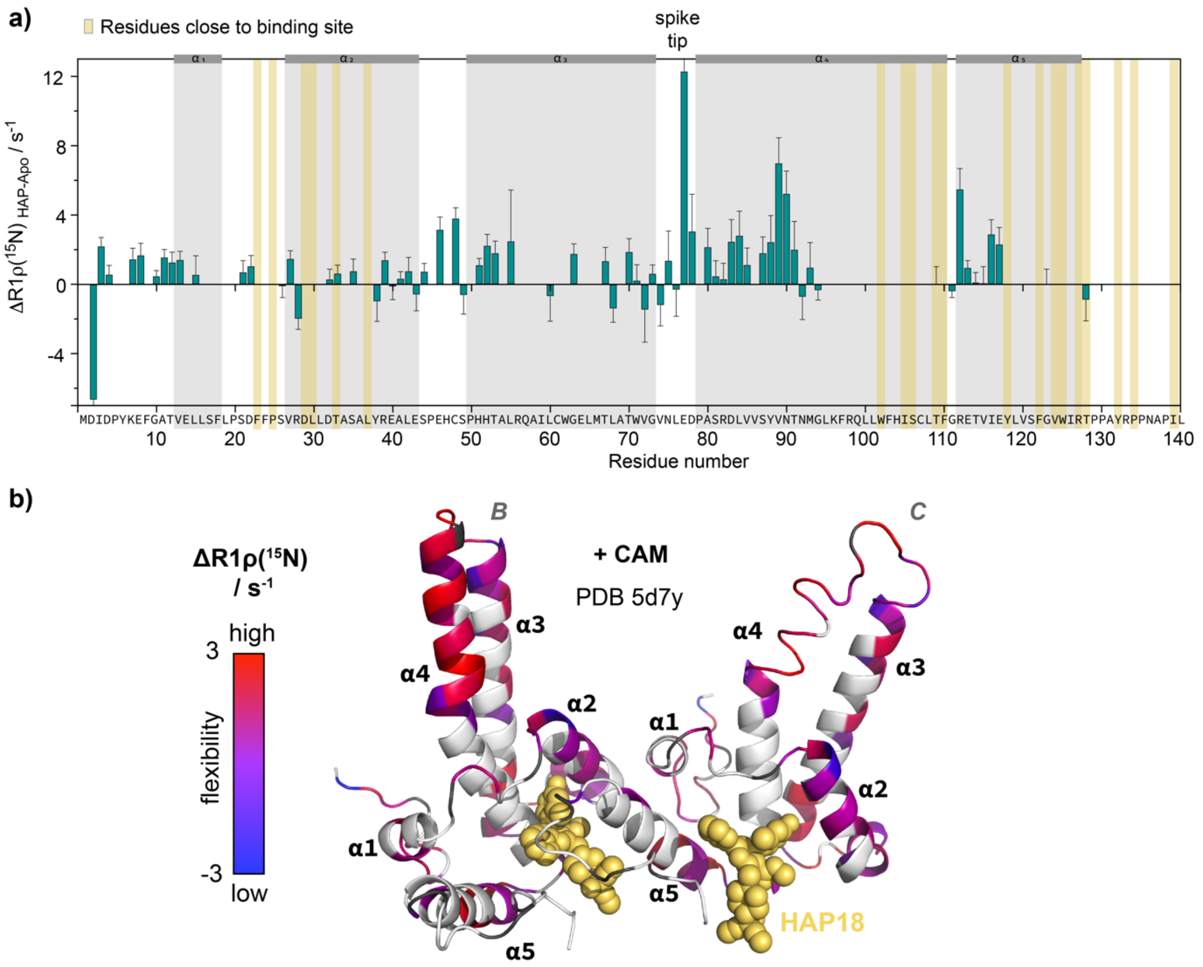

Supplementary Fig. 3a-b: a) Differences of $R_{1\rho}(^{15}N)$ rate constants measured at 80 kHz MAS and 13 kHz spin-lock field plotted for each residue upon JNJ-890 binding. n=1 independent experiments have been recorded. The bars represent fitted relaxation rate constants $R_{1\varrho}(^{15}N) \pm 2$ times the standard deviation (see material and Methods for details on $R_{1\varrho}(^{15}N)$ measurements). The error bars are derived from a bootstrap procedure. Source data are provided in the Source Data file. b) Differences in $R_{1\rho}$ relaxation parameters mapped on the Cp149 structure (PDB 5d7y[2] with HAP18 molecule shown in golden spheres). The color scale goes from red (more flexible upon JNJ-890 binding) to blue (more rigid).

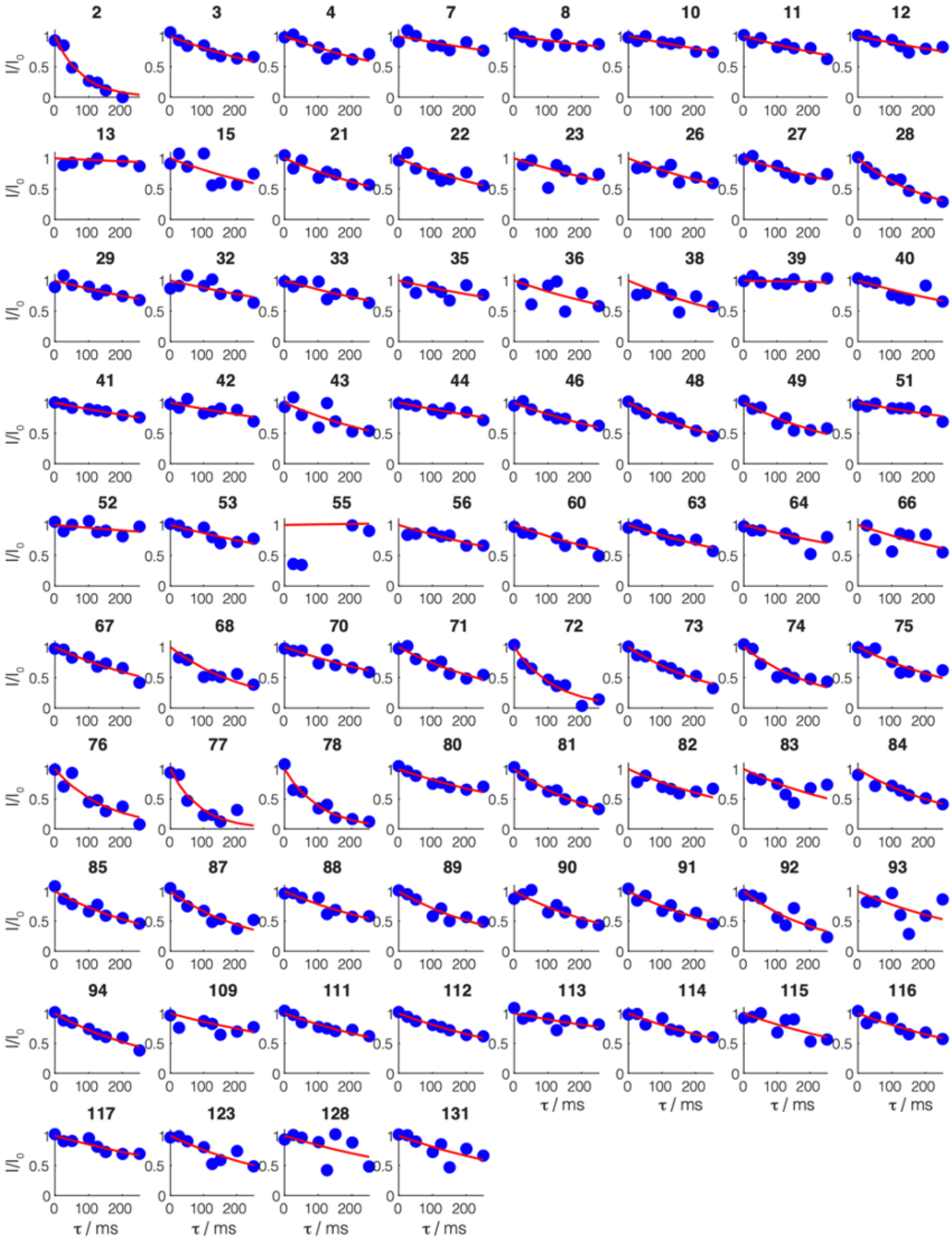

Supplementary Fig. 3c: Site-specific relaxation decay traces for $R_{1\rho}$(^{15}N) determination in Cp149 in absence of JNJ-890, using 3D hCANH experiments with a varying 13 kHz spin lock (1 μs to 251 ms) recorded in an 0.7 mm rotor at 80 kHz MAS frequency at a magnetic field of 850 MHz. The blue dots correspond to the intensities of the resonances extracted from the 3D hCANH spectra at different spin lock times. The corresponding mono-exponential fits are given as red lines. The numbers in bold correspond to the residue number. n=1 independent experiments have been recorded. Source data are provided in the Source Data file.

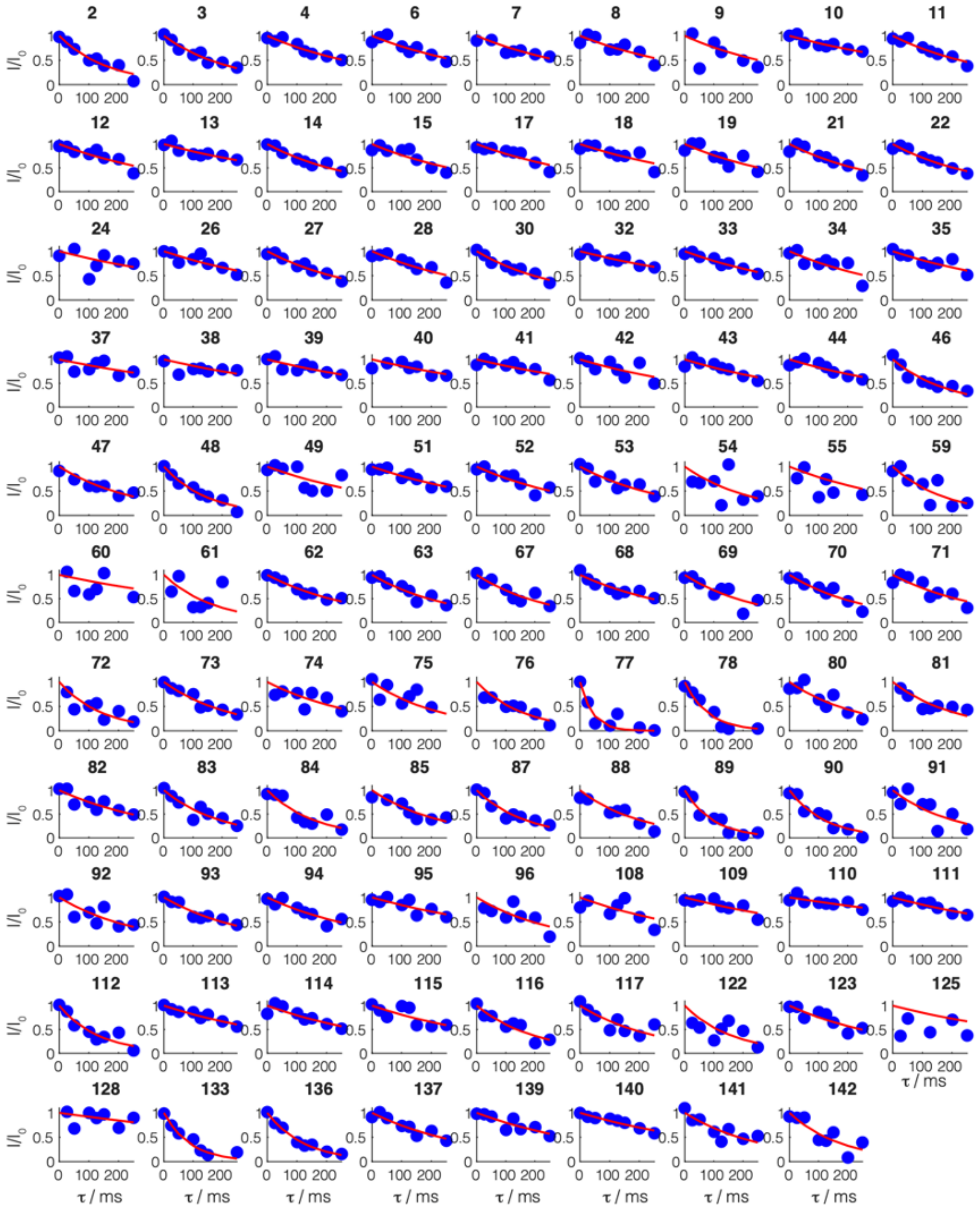

Supplementary Fig. 3d: Site-specific relaxation decay traces for $R_{1\rho}$(^{15}N) determination in Cp149 in presence of JNJ-890, using 3D hCANH experiments with a varying 13 kHz spin lock (1 µs to 251 ms) recorded in an 0.7 mm rotor at 80 kHz MAS frequency at a magnetic field of 850 MHz. The blue dots correspond to the intensities of the resonances extracted from the 3D hCANH spectra at different spin lock times. The corresponding mono-exponential fits are given as red lines. The numbers in bold correspond to the residue number. n=1 independent experiments have been recorded. Source data are provided in the Source Data file.

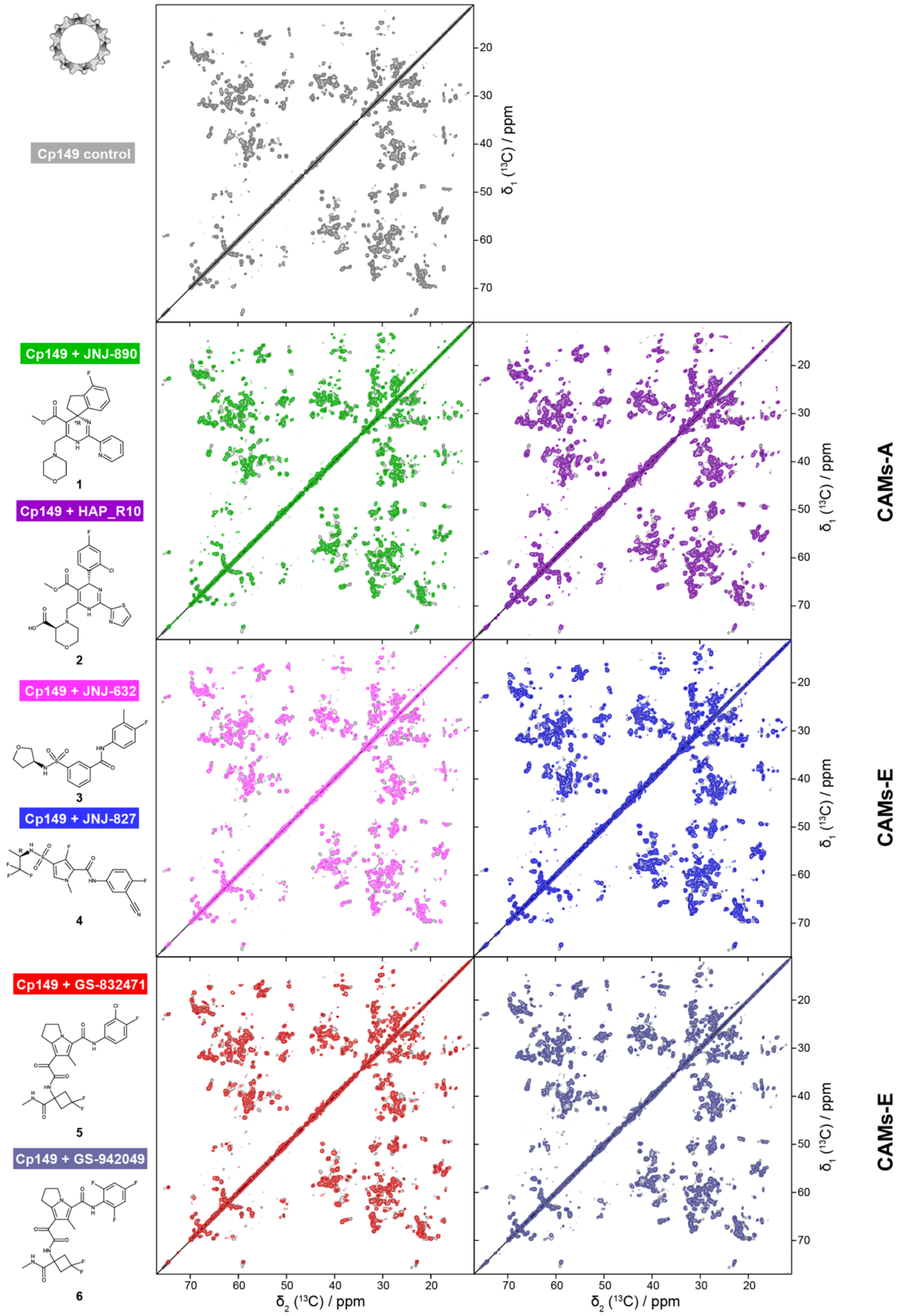

Supplementary Fig. 4a: NMR DARR spectra of all samples of ^{13}C-^{15}N Cp149 dimer reassembled in absence (grey, from reference[1]) and in presence of different CAM: JNJ-890 (green), HAP_R10 (GS-837886) (purple), JNJ-632 (pink), JNJ-827 (blue), GS-832471 (red) and GS-942049 (steel). The control is shown behind each spectrum in light grey. Corresponding EM pictures are shown in Figure 2 and chemical shift perturbation in Supplementary Fig. 5. n=1 independent experiments have been recorded.

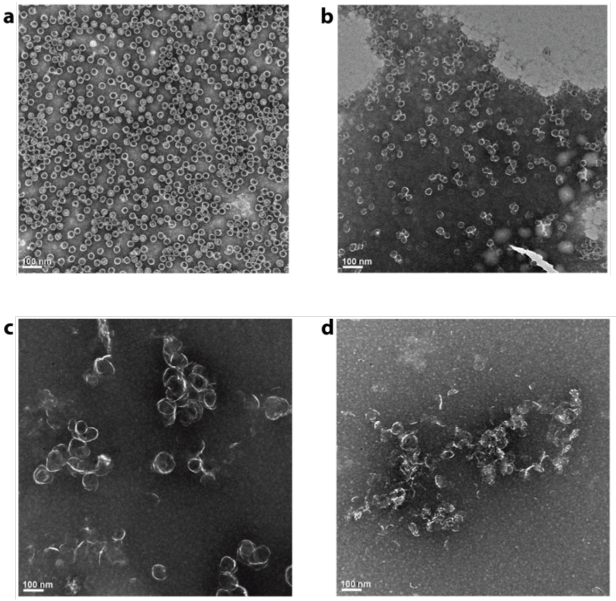

Supplementary Fig. 4b: Capsids do not disassemble on NMR sample preparation. In order to confirm that experimental conditions used during NMR sample preparation (low salt, 4 °C, ultracentrifugation) do not induce disassembly of the complexes into dimers, we analyzed representative resuspension of left-overs from sediments from NMR rotor filling and also a sediment removed from the rotor after the NMR experiment by negative staining EM. a) ^{13}C-^{15}N-Cp149+JNJ-632; b) ^{13}C-^{15}N-Cp149+JNJ-827; c) ^{13}C-^{15}N-Cp149+JNJ-890; d) ^{2}H-^{13}C-^{15}N-Cp149 +JNJ-890 (from NMR rotor). The micrographs reveal similar capsids or opened objects as the pictures taken from the samples before rotor filling and centrifugation. n=1 independent experiments have been recorded.

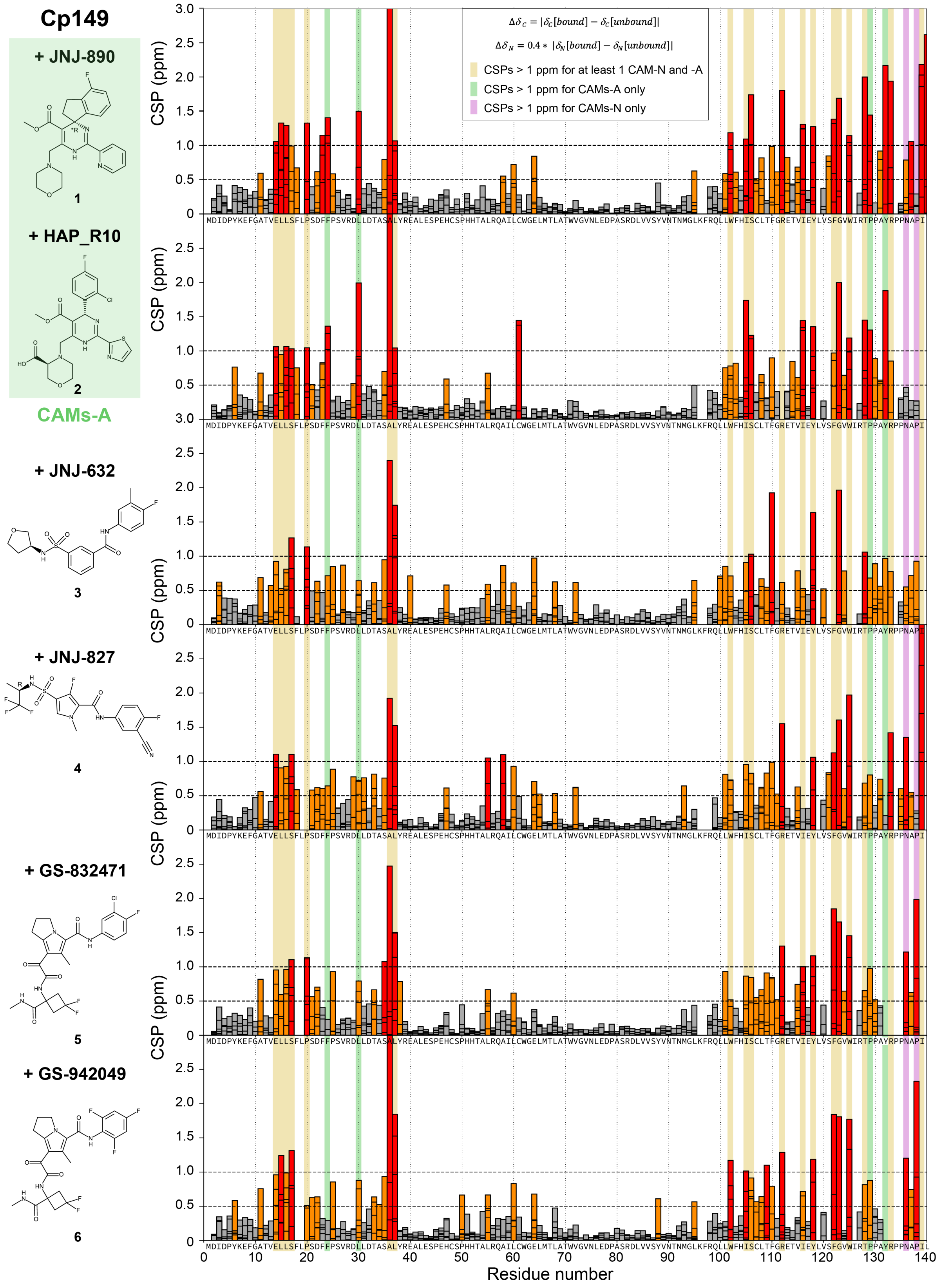

Supplementary Fig. 5a. Chemical shift perturbations induced by different CAMs on Cp149 capsids. CSPs were calculated for all assigned ^{15}N and ^{13}C nuclei using 2D and 3D spectra compared to the control sample without CAM. For ^{15}N CSPs, a factor of 0.4 was applied to account for the larger chemical shift range of ^{15}N (when compared to Cα). CSPs of the individual spins are shown as black lines inside the bars. Residues with medium

(0.5<CSP<1 ppm) and large (>1 ppm) CSPs are colored in orange and red, respectively. The CSPs (derived from the largest individual CSP observed for a given amino acid) mapped on the capsid structure are shown in Figure 2b. Residues the most affected by both CAMs-A and CAMs-E are highlighted in yellow, only by CAMs-A in green and only by CAMs-A in pink. n=1 independent experiments have been recorded. Individual values for CSPs are provided in the Source Data file.

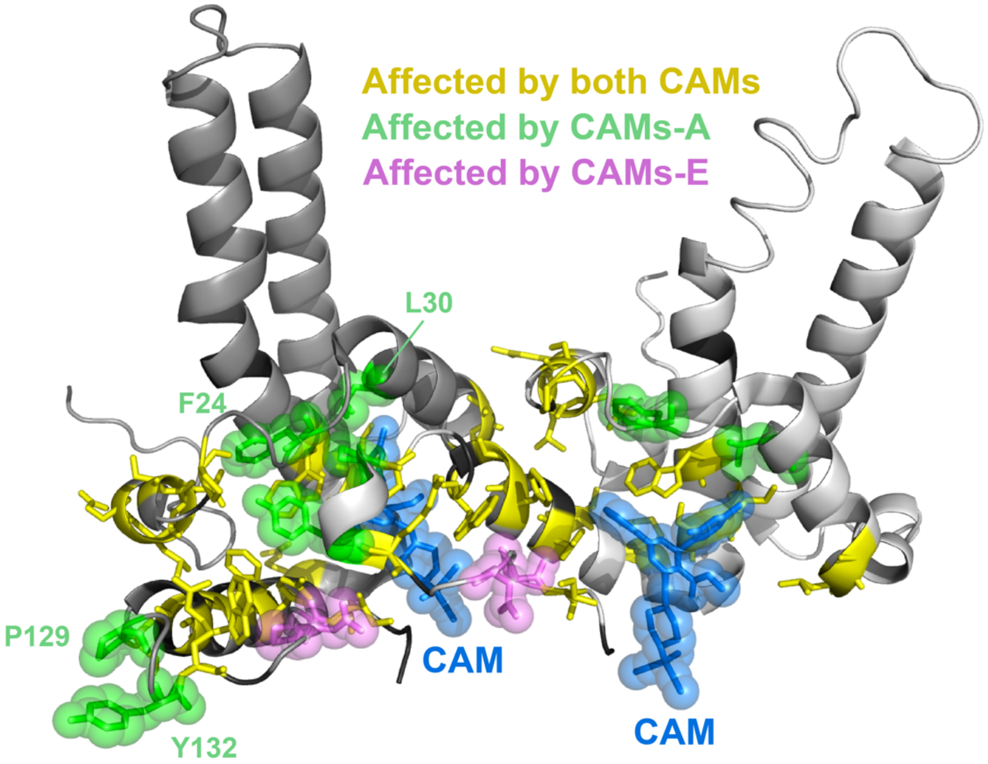

Supplementary Fig. 5b. CSPs induced by CAM-E and CAM-A compounds mapped on Cp structure. The structure highlights in yellow the residues which show CSPs for both CAM-E and CAM-A, and thus define the binding atoms. These are indeed rather similar between CAM-A and CAM-E. In pink are highlighted residues that show large CSPs only with CAM-E. In green are highlighted residues that show large CSPs only with CAM-A (residue numbers are labeled on one chain), pointing to residues sensitive to the change in lattice organization. The presence of these thus point to a CAM-A. The most straightforward way to distinguish by NMR CAM-E and CAM-A is however through the collapse of the up to fourfold peak multiples which are observed for T=4 icosahedral capsids. PDB used: 5d7y[2].

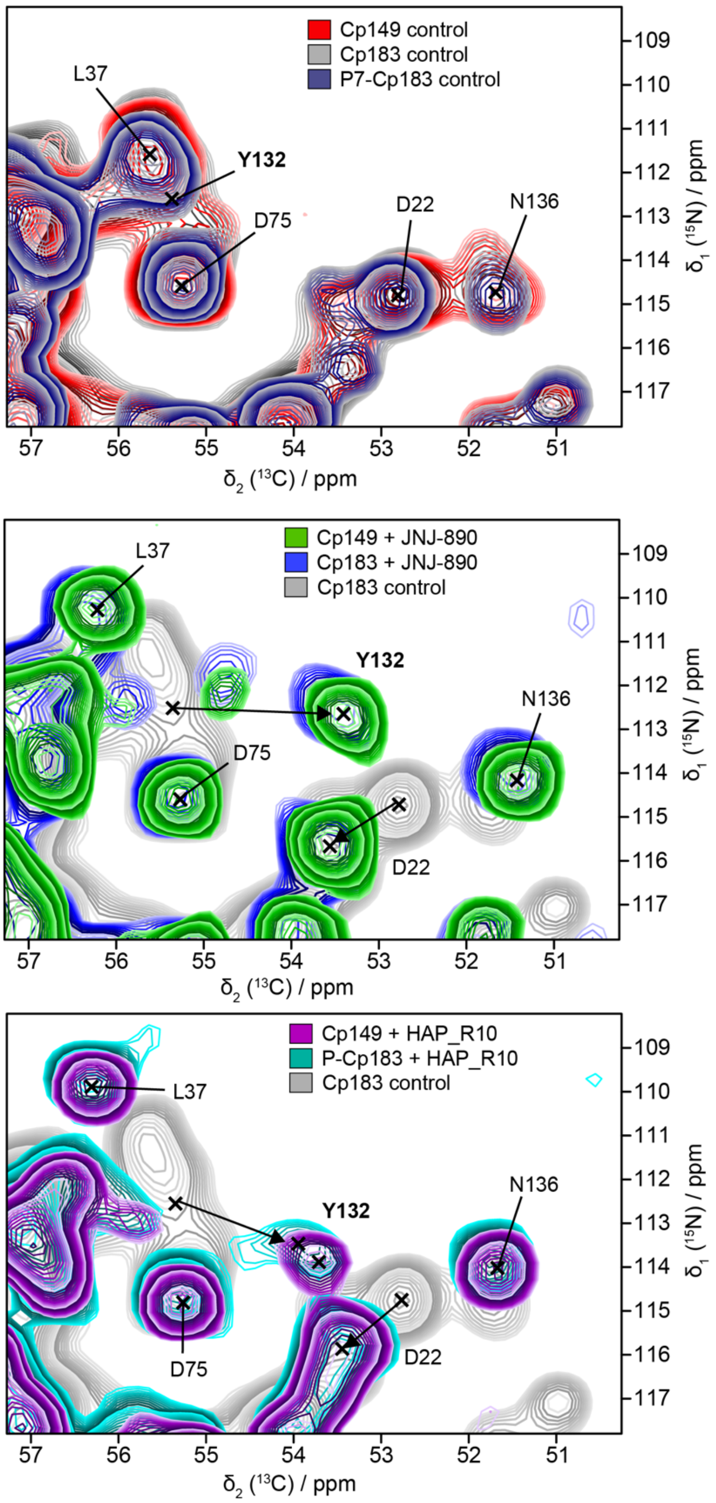

Supplementary Fig. 5c. Zoom on Y132 in 2D NCA spectra of representative Cp-CAM-A samples. Top panel: Overlay of Cp149 (red), Cp183 (grey) and P7-Cp183 (blue-grey) control samples. Middle panel: Overlay of Cp149 and Cp183 bound to JNJ-890, with Cp183 control (grey) as a reference. Bottom panel: Overlay of Cp149 and P7-Cp183 bound to HAP_R10, with Cp183 control (grey) as a reference. The chemical shifts of Y132 residue are strongly affected by the presence of both CAMs-A. n=1 independent experiments have been recorded.

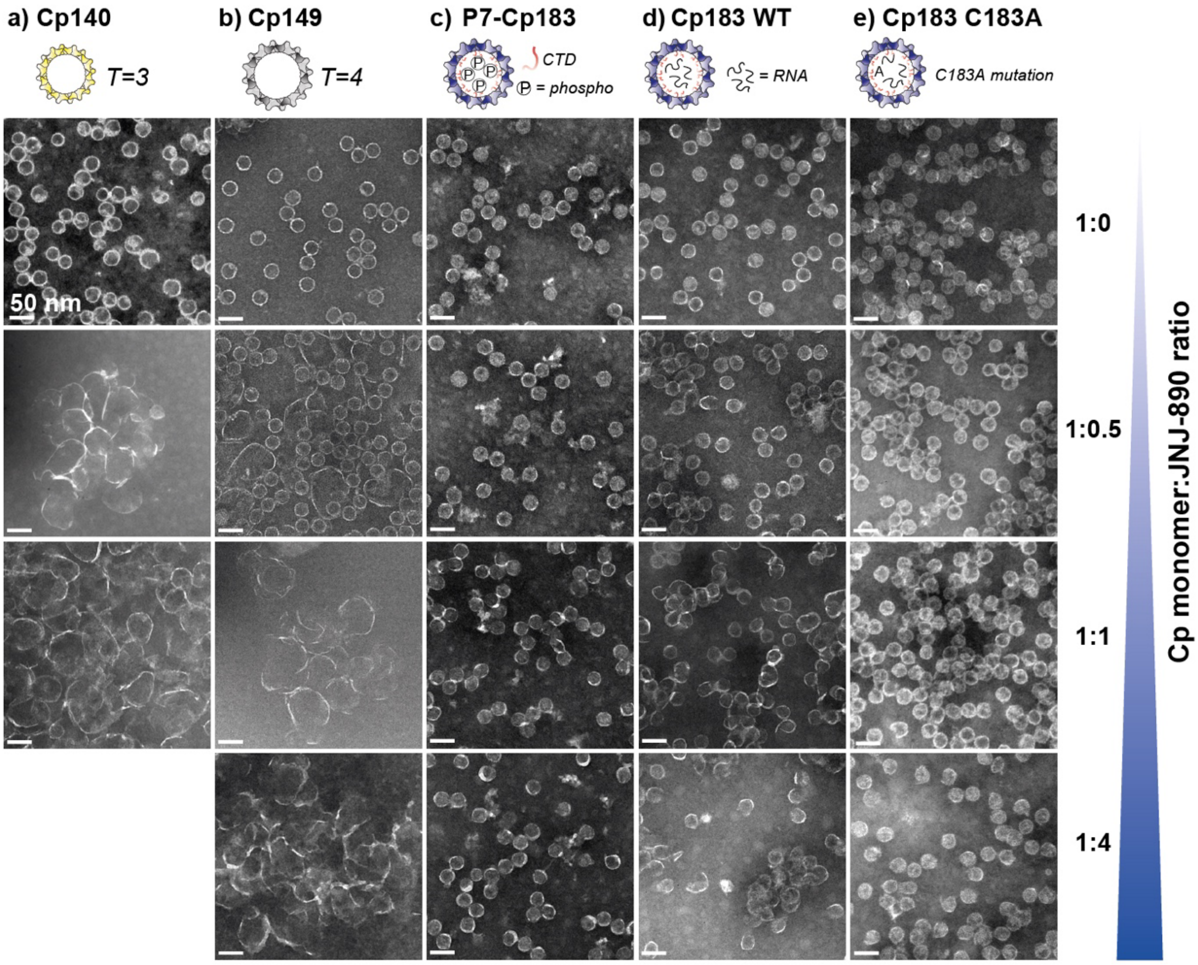

Supplementary Fig. 6: Impact of different JNJ-890 ratios on capsids. CAM-A at molar ratios from 0 to 4 molar equivalents were incubated with a) Cp140, b) Cp149, c) P7-Cp183 WT, d) Cp183 WT and e) Cp183 C183A mutant. Scale bars = 50 nm. The experiments were repeated independently at least 2 times with similar results, except for Cp140 which was done once.

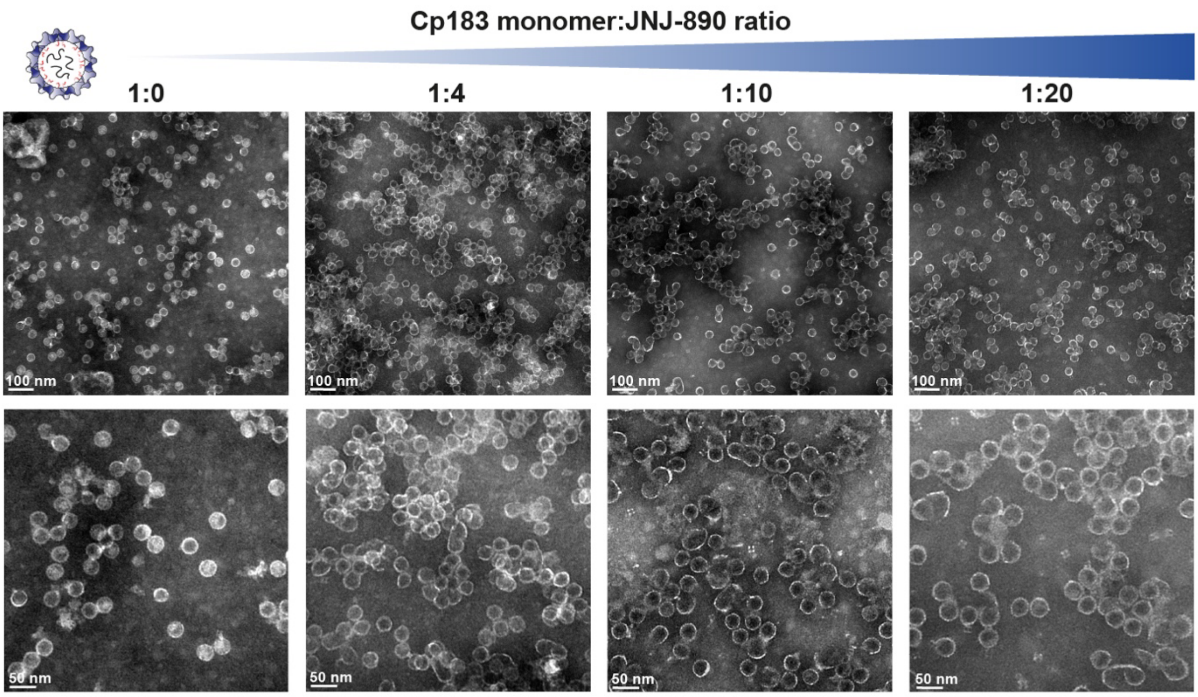

Supplementary Fig. 7: Impact of increasing ratios of JNJ-890 on wild-type Cp183. EM micrographs of Cp183 capsids control and incubated with 4, 10 and 20 molar equivalents of JNJ-890. Zooms are given for the four conditions. Even at high molar ratio (1:10 and 1:20 monomer:CAM-A), most capsids remain virtually closed. The experiments were repeated independently at least 2 times with similar results, except ratio 1:20 which was done once.

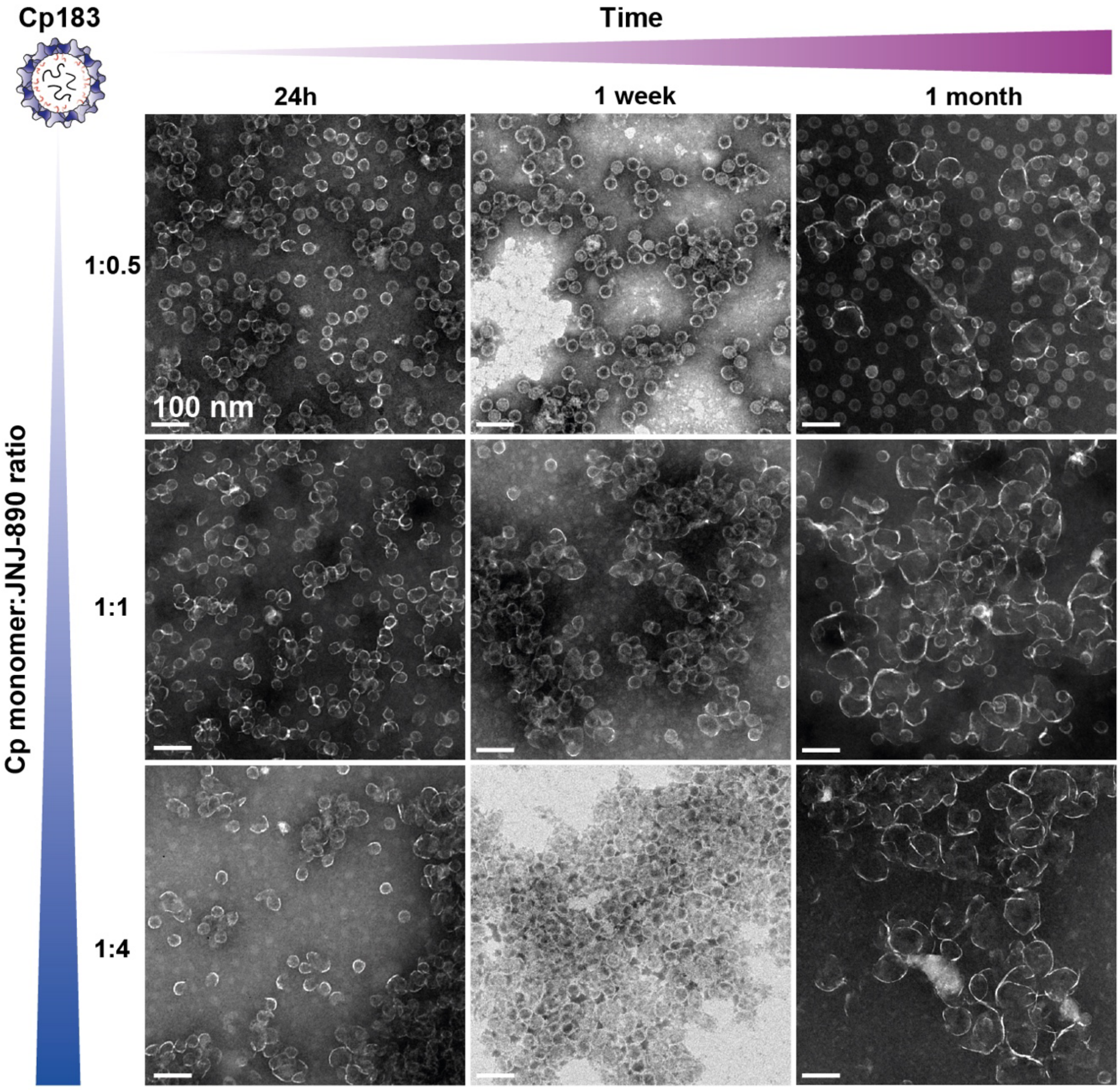

Supplementary Fig. 8: Impact of time on capsid's opening with different monomer:JNJ-890 ratio. The effect of CAM-A starts to be visible after 1 week, yet capsids are not all opened even after 1 month at the highest ratio. Scale bar = 100 nm. The experiment was done once.

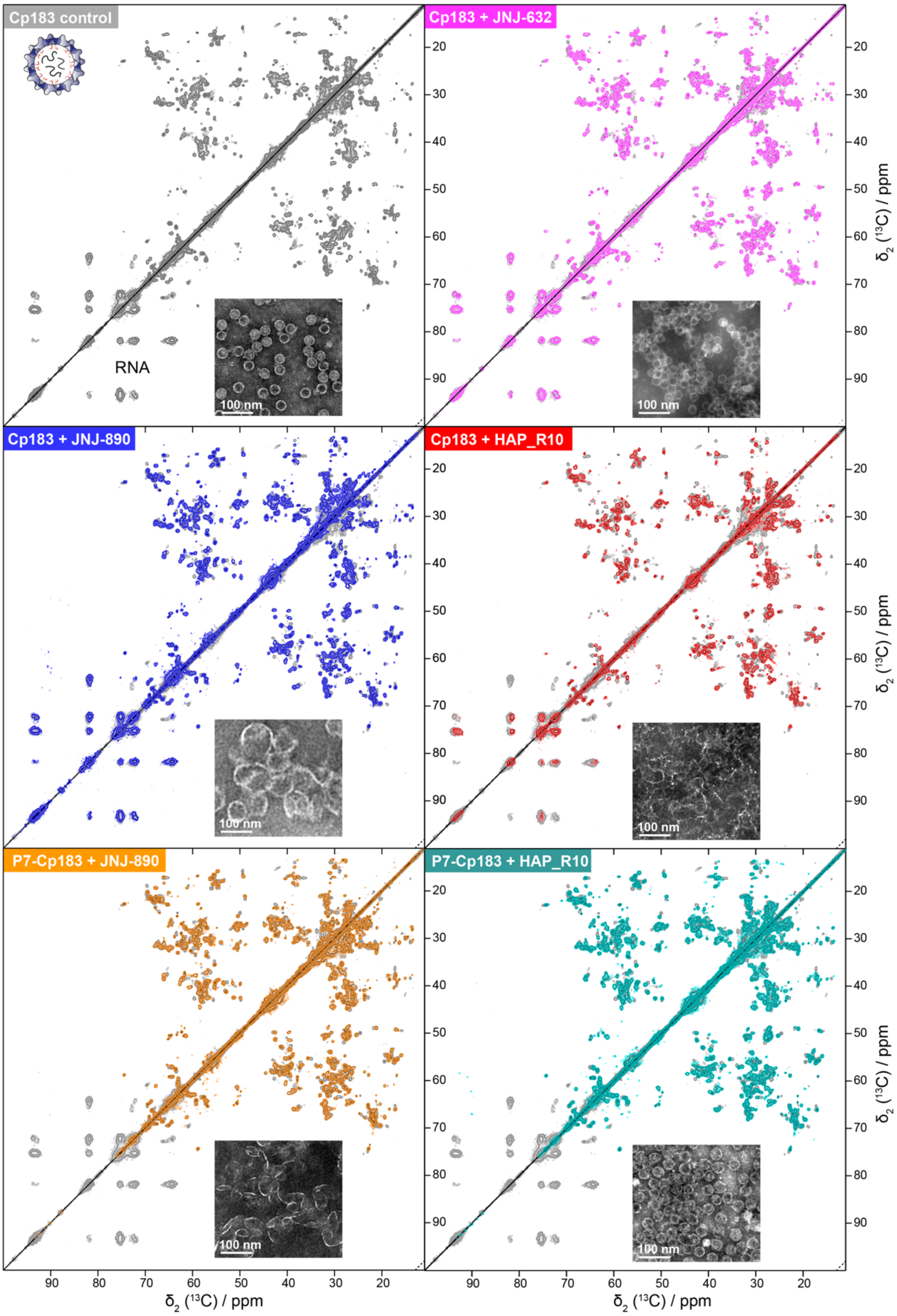

Supplementary Fig. 9: NMR aliphatic regions of DARR spectra and EM micrographs of Cp183 capsids. Absence of CAM (grey, from reference[1]); ^{13}C-^{15}N Cp183 capsids incubated with JNJ-632 (pink), JNJ-890 (blue) and HAP_R10 (red), ^{13}C-^{15}N P7-Cp183 capsids incubated with JNJ-890 (orange) and HAP_R10 (cyan). All capsids are in presence of 5 mM DTT. Capsids in absence of DTT yield same spectra, with the exception that they can reflect a different oxidation state of the cysteines. The EM pictures were taken on isotope-labeled samples, and thus n=1 independent experiments have been recorded.

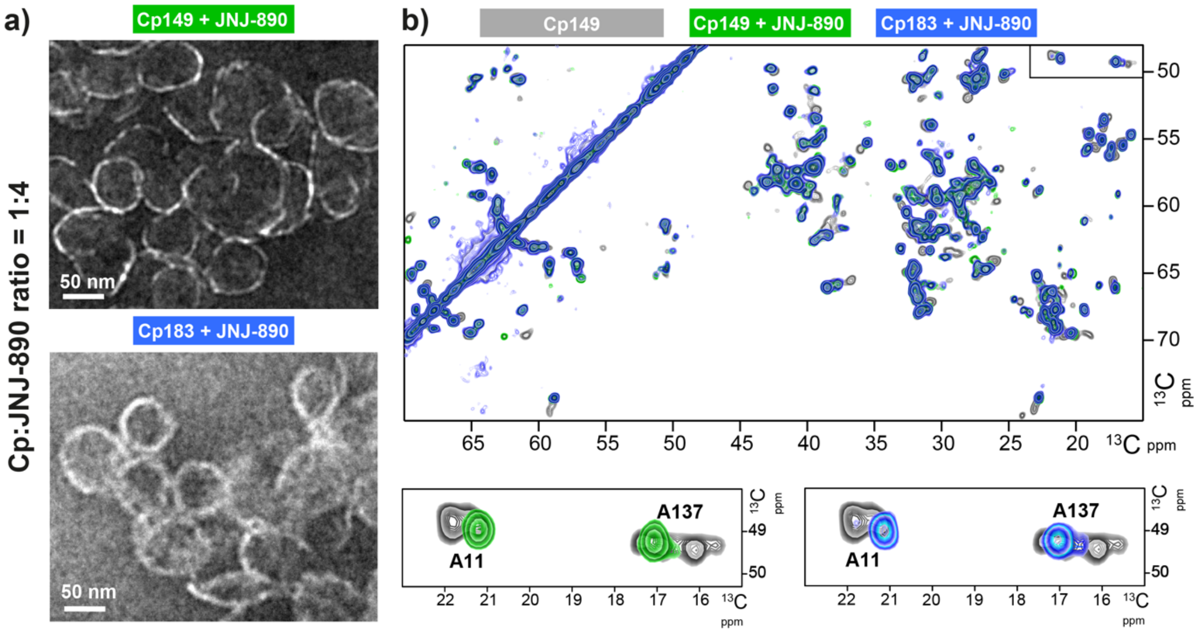

Supplementary Fig. 10: Impact of JNJ-890 HAP on Cp149 and Cp183 capsids. a) EM micrograph of Cp149 dimer reassembled with JNJ-890 (green) and of Cp183 preformed capsid incubated with JNJ-890 (blue). b) Region of 2D DARR spectra of Cp149 without CAM (in grey, from reference[1]), Cp149 reassembled with JNJ-890 (green) and Cp183 capsid with JNJ-890 (blue). Extracts from A11 and A137 region are shown below. The EM pictures were taken on isotope-labeled samples, and thus n=1 independent experiments have been recorded.

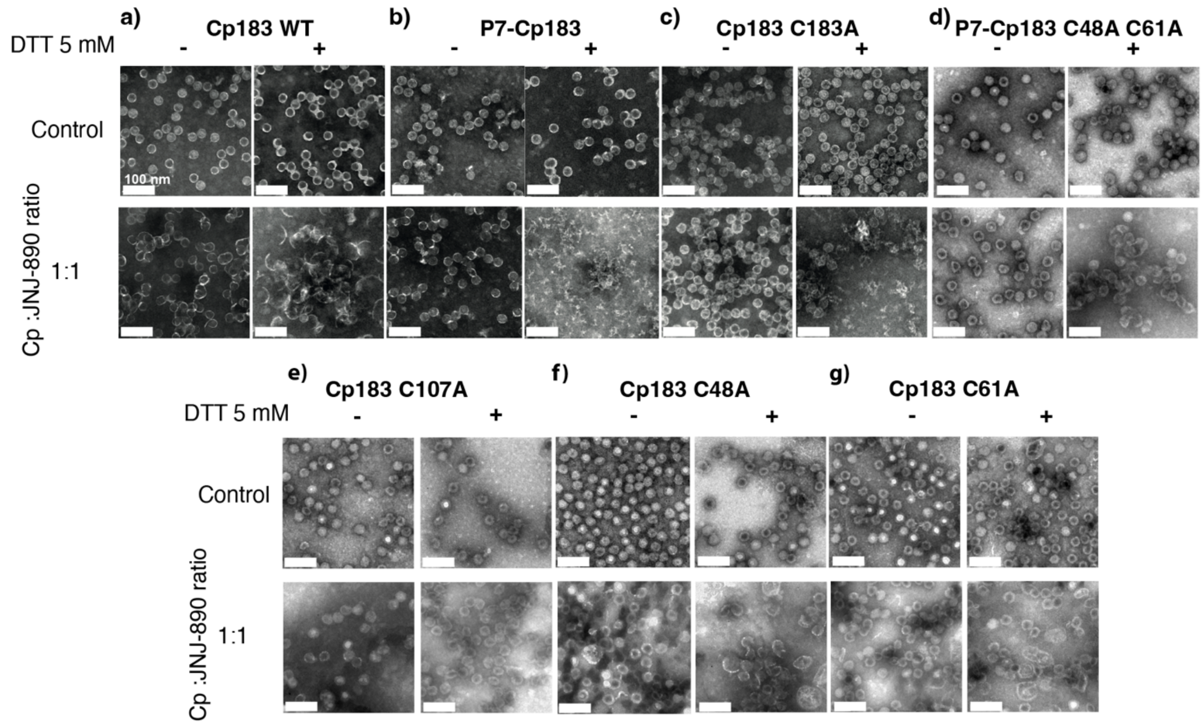

Supplementary Fig. 11: Impact of DTT on addition of 1 molar equivalent of JNJ-890. Scale bars 100 nm. It can be seen that for all forms, but C107A and C61A, DTT enhances Cp opening. The experiment was repeated independently at least 2 times with similar results for Cp183 WT, P7-Cp183 and Cp183 C183A, and n=1 independent experiments for the other mutants.

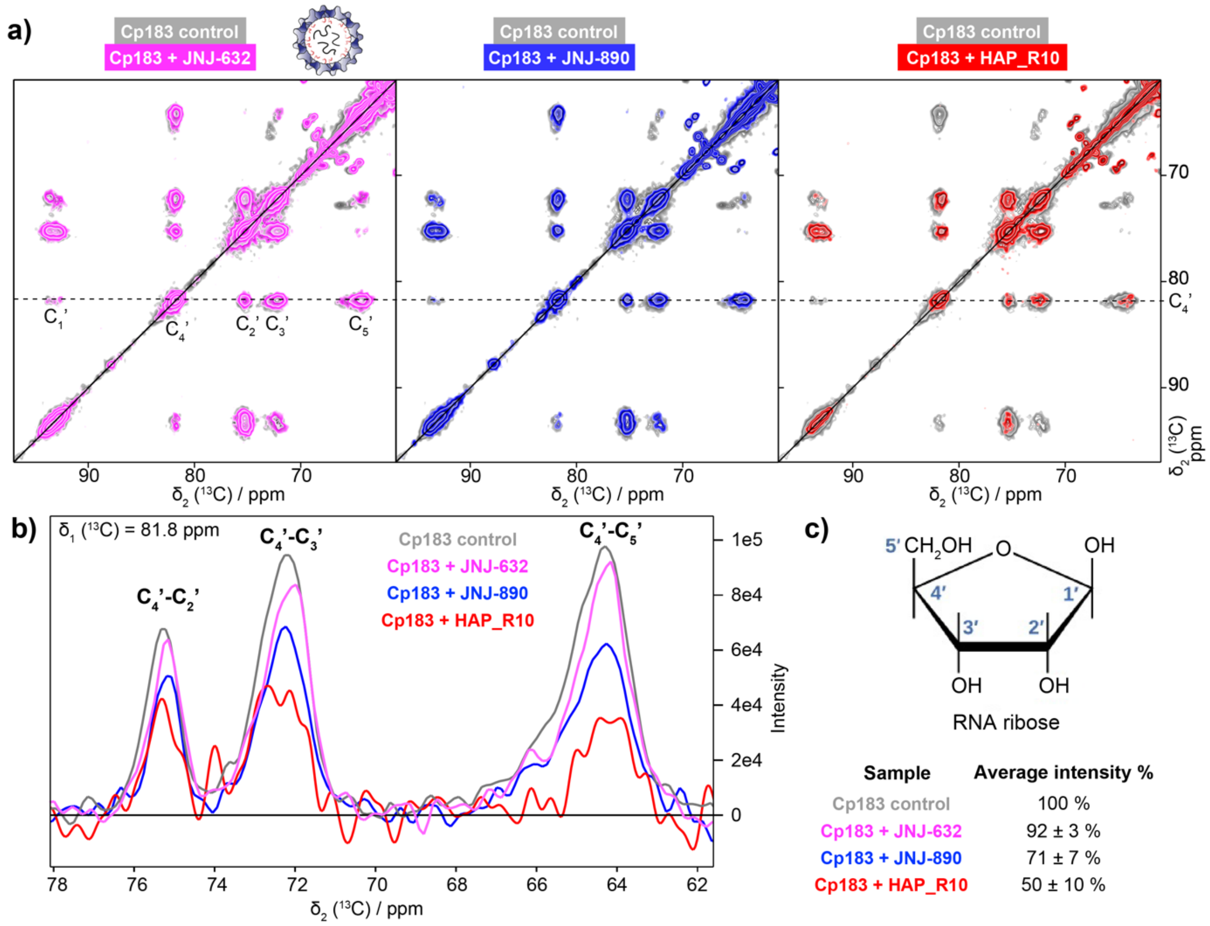

Supplementary Fig. 12: CAM-A leads to loss of RNA on Cp183 preformed capsids. a) Region from DARR spectra corresponding to nucleic acids overlayed for Cp183 apo capsids (grey, from reference[1]) and Cp183 capsids incubated at a 1:4 ratio Cp:CAM with JNJ-632 (pink), JNJ-890 (blue) and HAP_R10 (red). Corresponding EM micrographs are shown in Supplementary Fig. 9. b) 1D trace of the three major nucleic acids correlation signals extracted at 81.8 ppm (corresponding to C_4' carbon in ribose). Spectra intensities were calibrated on the protein signals. c) Representation of RNA ribose and table summarizing the average intensity of the CAMs-bound samples compared to Cp183 sample in absence of CAMs for the three RNA signals. The capsids investigated were purified including by a sucrose gradient (see Material and Methods section), and therefore carry no free RNA before CAM addition. Thus, the RNA observed in the reference and JNJ-632 capsids is the one from inside the capsid. After capsid opening by CAM-A, the capsids are directly ultracentrifuged into the rotor. During this step, free RNA would likely remain in the supernatant, or be too flexible to be observed in these spectra. The decay in signal intensity thus points to partial release of the packaged RNA into solution, while a part remains associated with Cp, but is not enclosed (protected) anymore. Since no nucleases are present in the solution, they are however not degraded. n=1 independent experiments have been recorded.

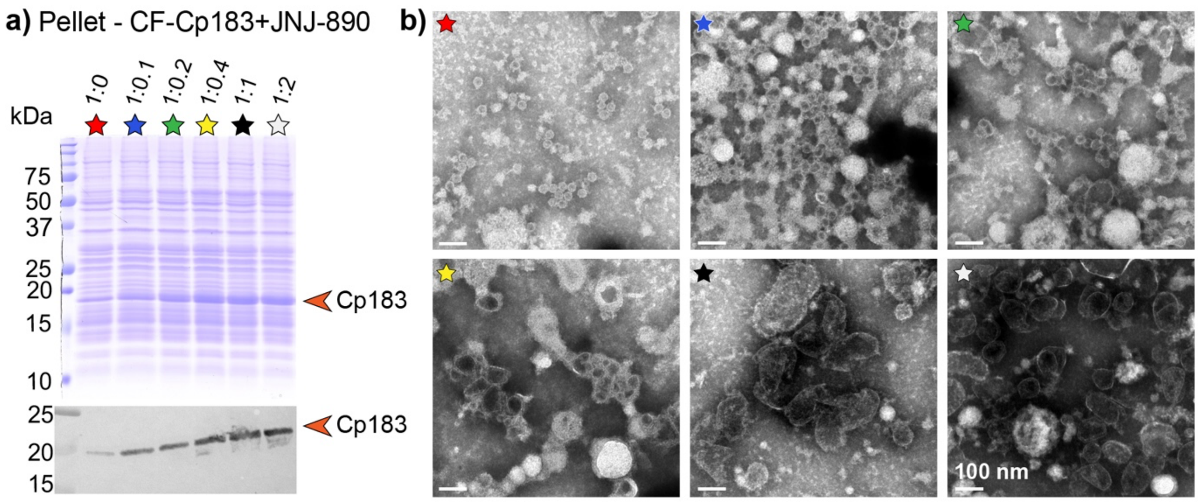

Supplementary Fig. 13: Capsid solubility and morphology of CF-Cp183 capsids synthesized in the presence of JNJ-890. a) SDS-PAGE and Western-Blot analyses of CF-Cp183 with increasing molar ratios of JNJ-890 (pellet fraction) (for uncropped gels see Source Files section). While in absence of JNJ-890 (red star), most Cp183 is soluble, after addition of CAM-A capsids are mostly found in the pellet. b) Negative-staining EM micrographs corresponding to the crude cell-free synthesis reactions, before centrifugation. Capsid's sizes start to increase from the ratio monomer:CAM-A 1:0.1, and capsids are almost fully open from a ratio 1:0.4 (yellow star). n=1 independent experiments have been recorded, except for ratio 1:0.4 which was repeated independently at least 2 times and yielded similar results.

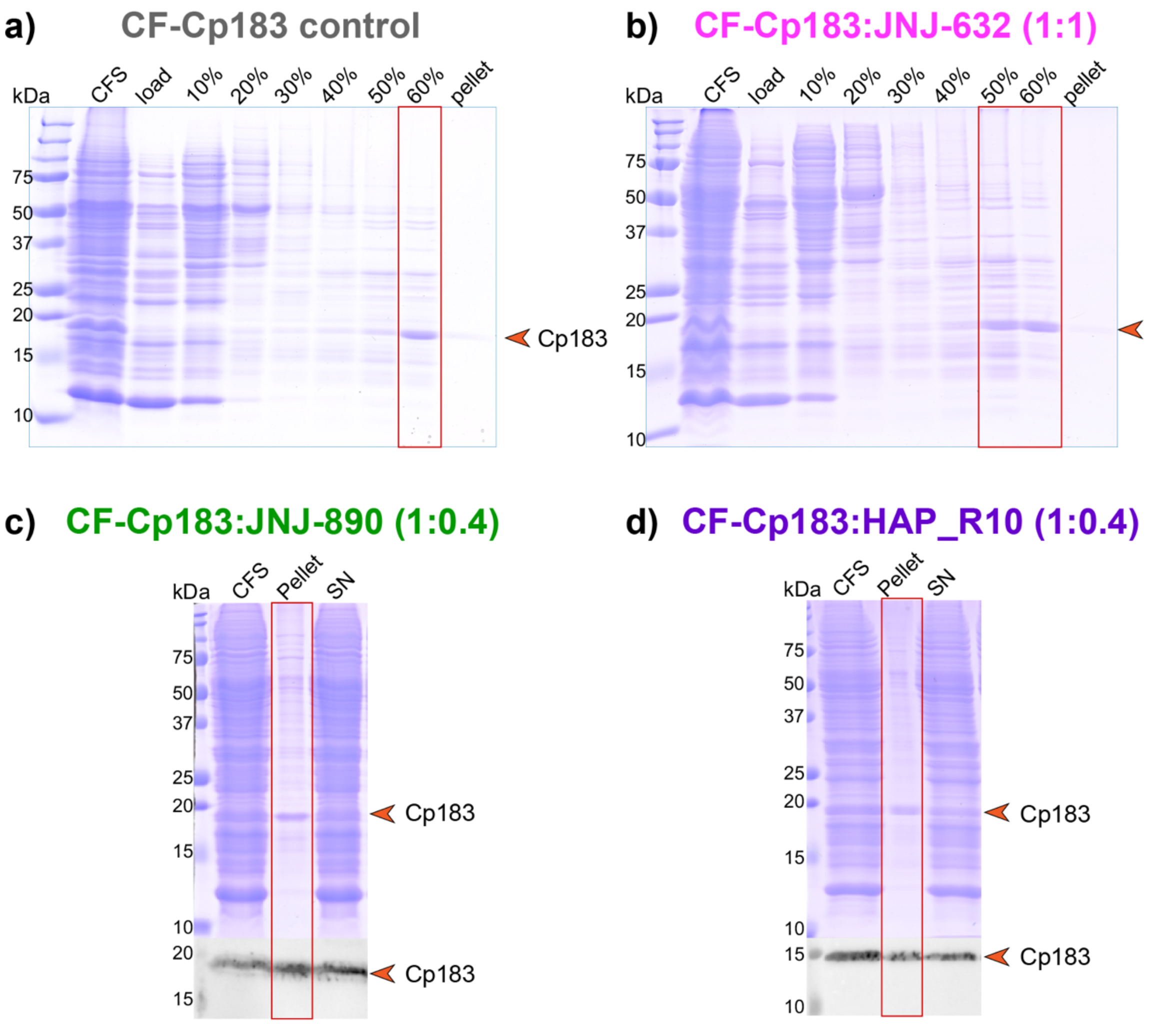

Supplementary Fig. 14: ^{2}H-^{13}C-^{15}N CF-Cp183 synthesis in absence or presence of different CAMs. SDS-PAGE of CF-Cp183 purification in presence of a) no CAM, b) 1 equivalent of JNJ-632, c) 0.4 equivalent of JNJ-890 and d) 0.4 equivalent of HAP_R10. For the control without CAM and the sample with CAM-E (JNJ-632), the capsids are mainly soluble and migrate on a sucrose gradient, while for both CAMs-A (JNJ-890 and HAP_R10), a large part of the protein sample is found in the pellet after centrifugation and can be directly sedimented into the NMR rotors. The fractions used to fill the 1.3 mm rotors are framed in red. Western-Blots are shown in panel c) and d). CFS: total cell-free sample; 10-60 %: sucrose gradient fractions; SN: supernatant. Corresponding EM pictures and hNH solid-state NMR spectra are shown in the main text in Figure 4. The experiments were repeated independently at least 2 times and yielded similar results. For uncropped gels see Source Files section.

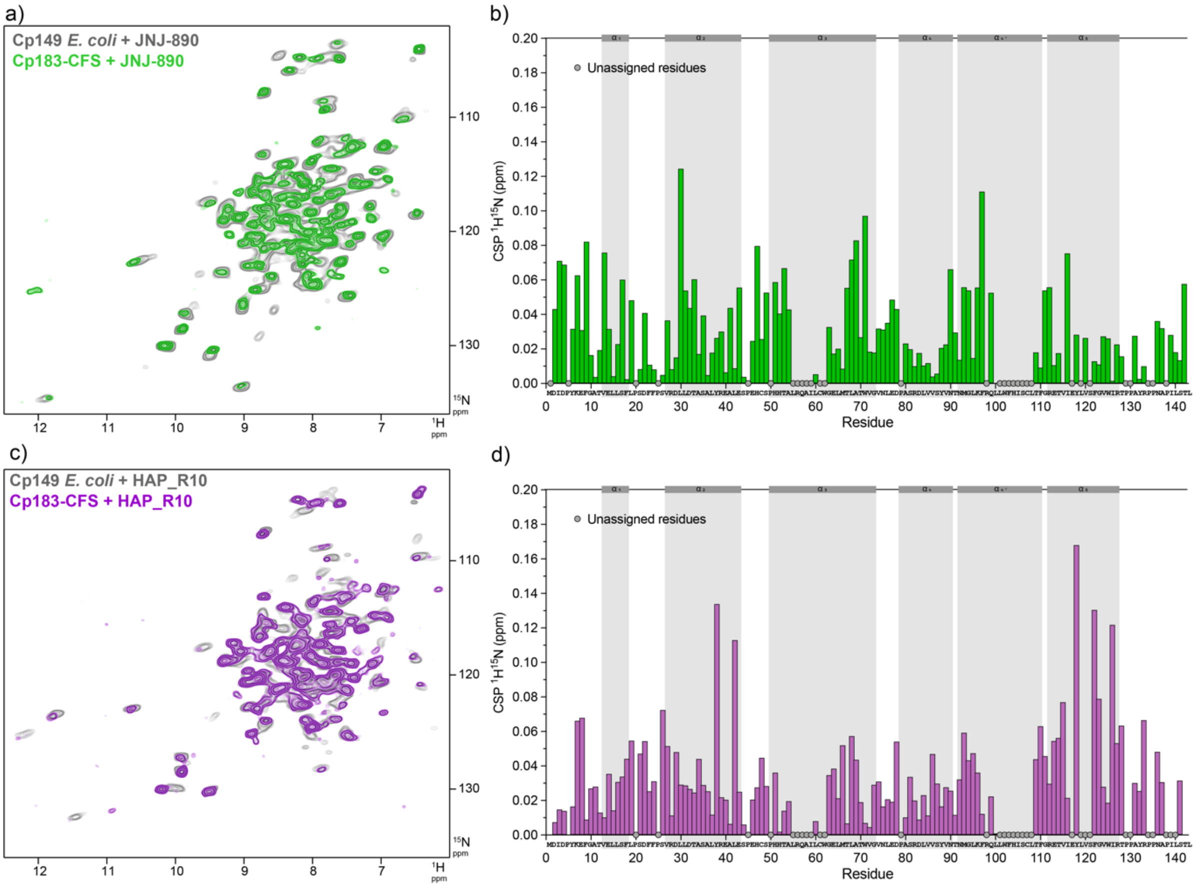

Supplementary Fig. 15: CSPs cell-free versus *E. coli* capsids in presence of CAMs-A. a) Overlay of 2D hNH spectra of Cp149 dimer produced in *E. coli* reassembled with JNJ-890 (grey) and CFS-Cp183 produced in presence of JNJ-890 (green). b) HN-CSP graph showing the differences between the two spectra. c) Overlay of 2D hNH spectra of Cp149 dimer produced in *E. coli* reassembled with HAP_R10 (grey) and CFS-Cp183 produced in presence of HAP_R10 (purple). d) HN-CSP graph showing the differences between the two spectra, which for the largest part are small (< 0.1 ppm), and none > 0.2 ppm. n=1 independent experiments have been recorded. Source data are provided in the Source Data file.

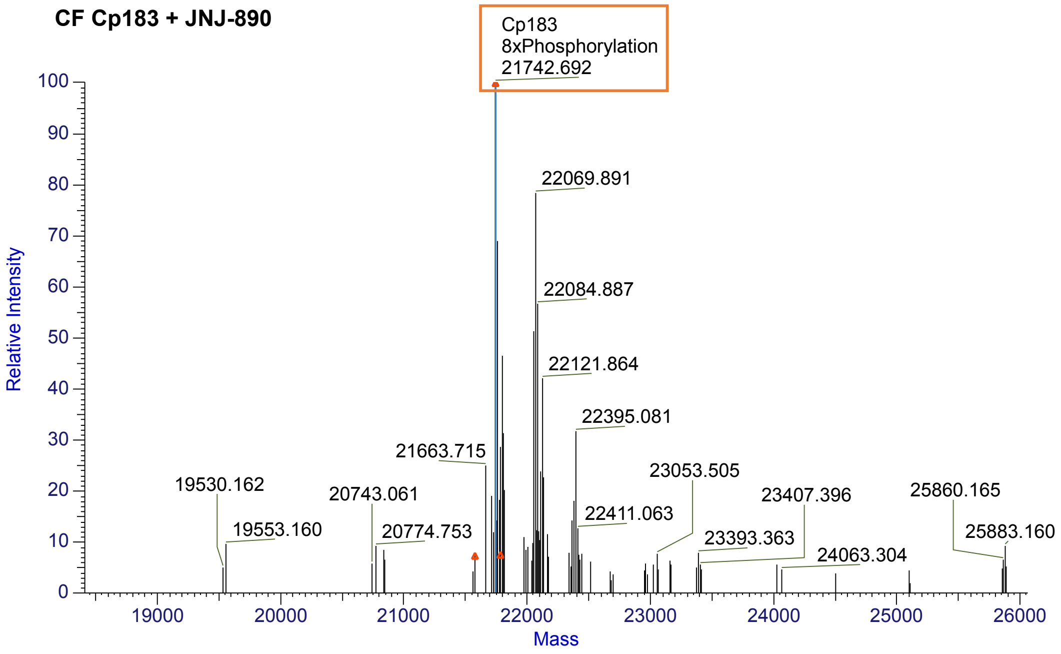

Supplementary Fig. 16: Mass spectrometry of unlabeled Cp183 produced in WG-CFPS in presence of 0.4 equivalents of JNJ-890. A mass of 21742 Da is observed, which corresponds to the presence of 8 phosphorylations (expected mass without phosphorylation = 21116 Da). n=1 independent experiments have been recorded.

Supplementary Table 1: Formula and molecular weights of CAMs-A and CAMs-E.

CAMs-A		CAMs-E	
**JNJ-890**<br>$C_{24}H_{25}F$ N_4O_3<br>436.4 g/mol	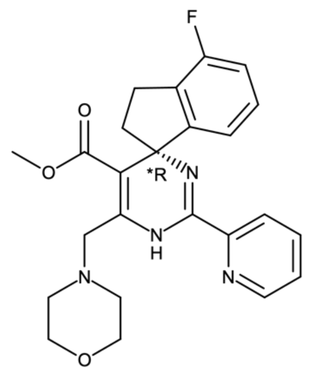<br>**1**	**JNJ-632**<br>$C_{18}H_9SF$ N_2O_4<br>378.4 g/mol	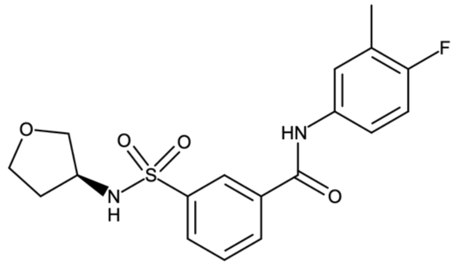<br>**3**
**HAP_R10** (GS-837886)<br>$C_{21}H_{20}F$ N_4O_5SCl<br>494.9 g/mol	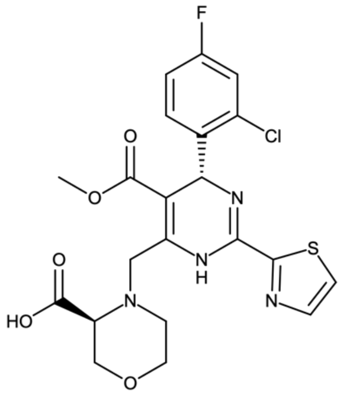<br>**2**	**JNJ-827**<br>$C_{16}H_{13}S$ $F_5N_4O_3$<br>436.3 g/mol	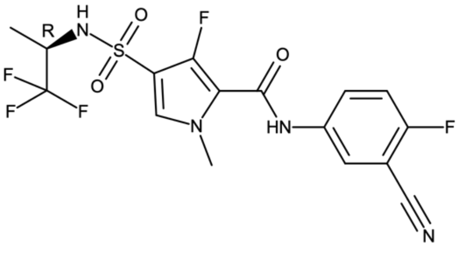<br>**4**
		**GS-832471**<br>$C_{23}H_{22}F_3$ N_4O_4Cl<br>510.9 g/mol	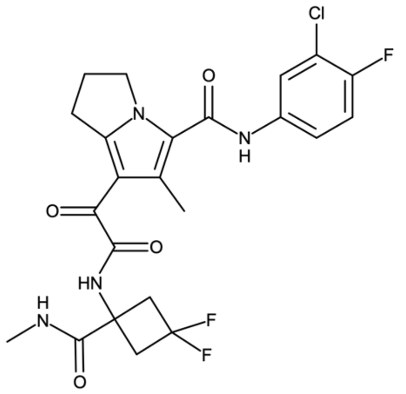<br>**5**
		**GS-942049**<br>$C_{23}H_{21}F_5$ N_4O_4<br>512.4 g/mol	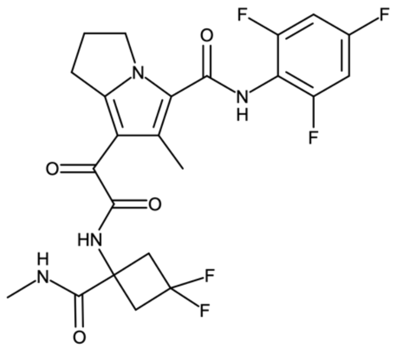<br>**6**

Supplementary Table 2: List of NMR samples used and experiments recorded. Colors used in the first column correspond to the colors used for the NMR spectra. All samples were devoid of Triton X-100 detergent[1] except Cp149 capsids + JNJ-632 as well as Cp183 with JNJ-632 and JNJ-890, for which the gel filtration step was not performed. NMR experimental details are described in Tables S3 to S5.

**Sample Name**	**Labeling**	**NMR experiments**	**System**	**Incubation**	**Figures**
Cp149 control	^{2}H-^{13}C-^{15}N	hNH, hCANH, hCONH, hcaCBcaNH, $R_{1\rho}(^{15}N)$ hCANH	*E. coli*	Cp149 dimer reassembled with 150 mM NaCl	1f, S1, S3, S15
Cp149 control	^{13}C-^{15}N	DARR, NCA, NCO, NCACX, NCOCX, CANCO	*E. coli*		1d, 2, S4, S5a-c
Cp149 + JNJ-890	^{2}H-^{13}C-^{15}N	hNH, hCANH, hCONH, hcaCBcaNH, hCAcoNH, $R_{1\rho}(^{15}N)$ hCANH	*E. coli*	Cp149 dimer reassembled with CAMs for 24h at RT with 150 mM NaCl	1f, S1, S3, S15
Cp149 + JNJ-890	^{13}C-^{15}N	DARR, NCA, NCO, NCACX, NCOCX, CANCO	*E. coli*		1d, 2, S2a, S4, S5a-c, S10
Cp149 + HAP_R10	^{2}H-^{13}C-^{15}N	hNH, hCANH, hCONH, hcaCBcaNH, hCAcoNH	*E. coli*		S15
Cp149 + HAP_R10	^{13}C-^{15}N	DARR, NCA, NCO, NCACX	*E. coli*		2, 3, S4, S5a-c
Cp149 + GS-942049	^{13}C-^{15}N	DARR, NCA, NCACX	*E. coli*		2, S4, S5a
Cp149 + GS-832471	^{13}C-^{15}N	DARR, NCA, NCACX	*E. coli*		2, S4, S5a
Cp149 + JNJ-827	^{13}C-^{15}N	DARR, NCA, NCO, NCACX, NCOCX, CANCO	*E. coli*		2, S4, S5a
Cp149 + JNJ-632	^{13}C-^{15}N	DARR, NCA, NCO, CANCO	*E. coli*		2, S2b, S4, S5a
Cp149 capsids + JNJ-890	^{13}C-^{15}N	DARR, NCA, NCO	*E. coli*	Cp149 purified capsids incubated with 4 eq. CAMs 2h at 37 °C	S2a
Cp149 capsids + JNJ-632	^{13}C-^{15}N	DARR, NCA, NCO, NCACX, NCOCX	*E. coli*		S2b
Cp183 control	^{13}C-^{15}N	DARR, NCA	*E. coli*		S5c, S9, S12
Cp183 + JNJ-632	^{13}C-^{15}N	DARR, NCA, NCO	*E. coli*	Cp183 purified capsids incubated with 4 eq. CAMs 2h at 37 °C	S9, S12
Cp183 + JNJ-890	^{13}C-^{15}N	DARR, NCA, NCO	*E. coli*		S5c, S9, S10, S12
Cp183 + HAP_R10	^{13}C-^{15}N	DARR	*E. coli*		S9, S12
P7-Cp183 + JNJ-890	^{13}C-^{15}N	DARR	*E. coli*		S9
P7-Cp183 + HAP_R10	^{13}C-^{15}N	DARR, NCA, NCO, NCACX	*E. coli*		3, S5c, S9
CF-Cp183 control	^{2}H-^{13}C-^{15}N	hNH, HP-CP	Cell-free		4
CF-Cp183 + JNJ-632	^{2}H-^{13}C-^{15}N	hNH, HP-CP	Cell-free	1 eq added upon synthesis	4
CF-Cp183 + JNJ-890	^{2}H-^{13}C-^{15}N	hNH, HP-CP	Cell-free	0.4 eq. added upon synthesis, sedimented from the pellet	4, S15
CF-Cp183 + HAP_R10	^{2}H-^{13}C-^{15}N	hNH, HP-CP	Cell-free		4, S15

Supplementary Table 3: Experimental parameters for ssNMR experiments using ^{13}C-detection at 17.5 kHz MAS frequency. Experiments were run on a 3.2 mm probe on a 800 MHz spectrometer at a temperature estimated around 4 °C. 90° pulses were: 2.5 µs in ^{1}H, 5 µs in ^{13}C and 6.1 µs in ^{15}N. For CP frequencies and transfer times, the average values with the standard deviation are given, which were calculated amongst the concerned samples as listed in Supplementary Table 2. For increments, sw (spectral width) and acquisition times of 3D spectra as well as for the number of scans and experimental time, the range of the different values used are indicated.

**Experiment**	**DARR**	**NCA**	**NCO**	**NCACX**	**NCOCX**	**CANCO**
MAS [kHz]	17.5					
Field [T]	18.8					
**Transfer I**	HC-CP	HN-CP	HN-CP	HN-CP	HN-CP	HC-CP
^{1}H field [kHz]	65 ± 1	55.4 ± 0.4				65 ± 1
X field [kHz]	50 (^{13}C)	41 (^{15}N)				50 (^{13}C)
Shape	Tangent ^{1}H					
^{13}C carrier [ppm]	58.6					
time [ms]	0.80 ± 0.15	0.85 ± 0.15				0.80 ± 0.15
**Transfer II**	DARR	NCa-CP	NCO-CP	NCa-CP	NCO-CP	CaN-CP
Field [kHz]	17.5 (^{1}H)	6 (^{13}C)				
Field [kHz] (^{15}N)		11.2 ± 0.2	11.8 ± 0.3	11.2 ± 0.2	11.8 ± 0.3	11.2 ± 0.2
Shape	-	Tangent ^{13}C				
^{13}C carrier [ppm]	100	58.6	177	58.6	177	58.6
time [ms]	20	9.7 ± 0.7	8 ± 2	9.7 ± 0.7	6.5 ± 1.0	9.7 ± 0.7
**Transfer III**				DARR	DARR	NCO-CP
Field [kHz]				17.5 (^{1}H)	17.5 (^{1}H)	6 (^{13}C) / 11.8 ± 0.3 (^{15}N)
Shape				-	-	Tangent ^{13}C
Carrier [ppm]				58.6	177	177
time [ms]				70	30	6.5 ± 1.0
t_1 increments	2560	1344	1344	74-90	32-50	38-70
sw (t_1) [ppm]	466	771	771	35 (^{13}Ca)	15 (^{13}CO)	40-60 (^{15}N)
Acq. time (t_1) [ms]	13.7	10.8	10.8	5.2 - 6.3	9.1	5.3 - 7.2
t_2 increments	3072	2304	2304	44-58	34-50	66-80
sw (t_2) [ppm]	466	497	497	40-50 (^{15}N)	40-42 (^{15}N)	32-40 (^{13}Ca)
Acq. time (t_2) [ms]	16.4	11.5	11.5	5.4-7.2	5.0-7.7	5.0-5.4
t_3 increments				2304	2304	2304
sw (t_3) [ppm]				497	497	497
Acq. time (t_3) [ms]				11.5	11.5	11.5
^{1}H decoupling during acq.	SPINAL64					
Field [kHz]	90					
Interscan delay [s]	2.6	2.6	2.6	2.6	2.3	3
Number of scans	8-16	8-16	8-16	16-32	16-64	16
Measurement time	15-30h	8-16h	8-16h	2-3 days	1-3 days	1-3 days

Supplementary Table 4: Experimental parameters for solid-state NMR experiments using ^{1}H-detection at 60 kHz MAS frequency. Experiments were run on a 1.3 mm probe at a temperature estimated between 20 and 25 °C. WALTZ16 was used for ^{1}H, ^{13}C and ^{15}N decoupling. Sw stands for spectra width. Carriers for ^{1}H and ^{15}N were at 4.8 and 118 ppm respectively.

**Samples** ^{2}H-^{13}C-^{15}N	Cp149 control<br>**Cp149 + JNJ-890**<br>**Cp149 + HAP_R10**					CF-Cp183 control<br>**CF-Cp183 + JNJ-632**<br>**CF-Cp183 + JNJ-890**<br>**CF-Cp183 + HAP_R10**	
**Experiment**	**CP hNH 2D**	**hCANH 3D**	**hCONH 3D**	**hCAcoNH 3D**	**hcaCBcaNH 3D**	**CP hNH 2D**	**HP-CP 1D**
Field / T	18.8					18.8	11.7
MAS / kHz	60					60	55-60
90° pulse (µs)	2.5 (^{1}H) / 4 (^{13}C) / 4 (^{15}N)					2.5(^{1}H)/ 4(^{13}C)/ 4(^{15}N)	2.5(^{1}H)/ 5(^{31}P)/
**Transfer I**	**HN CP**	**HC CP**				**HN CP**	**HP CP**
Field / kHz	43 (^{1}H)/ 17 (^{15}N)	25 (^{1}H) / 35 (^{13}C)				43 (^{1}H)/ 17 (^{15}N)	100 (^{1}H)/ 40 (^{31}P)
Shape	Tangent ^{1}H	Tangent ^{1}H				Tangent ^{1}H	
Carrier ^{13}C / ppm	-	56	178	56	56	-	-
Time / ms	~0.7	~4	2.5	~4	~4	0.9	1
**Transfer II**	**NH CP**	**CaN CP**	**CON CP**	**DREAM**	**CA-CB scalar**	**NH CP**	-
Field / kHz	43 (^{1}H)/ 17 (^{15}N)	25 (^{15}N) / 35 (^{13}C)	25 (^{15}N) / 35 (^{13}C)	30 (^{13}C)	-	43 (^{1}H)/ 17 (^{15}N)	-
Shape	Tangent ^{1}H	Tangent ^{13}C	Tangent ^{13}C	Tangent ^{13}C	-	Tangent ^{1}H	-
Carrier ^{13}C / ppm	-	56	178	178	42	-	-
Time / ms	~0.8	~8	~10	~10	~4	0.9	-
**Transfer III**	-	**NH CP**		**CaN CP**	**CaN CP**	-	-
Field / kHz	-	43 (^{1}H)/ 17 (^{15}N)		25 (^{15}N) / 35 (^{13}C)	25 (^{15}N) / 35 (^{13}C)	-	-
Shape	-	Tangent ^{1}H		Tangent ^{13}C	Tangent ^{13}C	-	-
Carrier ^{13}C / ppm	-	-	-	56	56	-	-
Time / ms	-	~0.8	~0.8	~8	~8	-	-
**Transfer IV**	-	-	-	**NH CP**	**NH CP**	-	-
Field / kHz	-	-	-	43 (^{1}H)/ 17 (^{15}N)	43 (^{1}H)/ 17 (^{15}N)	-	-
Shape	-	-	-	Tangent ^{1}H	Tangent ^{1}H	-	-
Time / ms	-	-	-	~0.8	~0.8	-	-
t1 increments	160 (^{15}N)	50-80 (^{13}C)	48-60 (^{13}C)	75 (^{13}C)	30-40 (^{15}N)	320	-
sw (t1) / ppm	40	22-30	14-16	30	24-35	80	-
Acq time (t1) / ms	24.7	~7	~10	6.2	~8	24.7	10.2
t2 increments	2048	32-58 (^{15}N)	40-64 (^{15}N)	32 (^{15}N)	~100 (^{13}C)	2048	-
sw (t2) / ppm	100	24-40	30-40	24	50-55	97.7	-
Acq time (t2) / ms	12.9	~9	~10	8.2	~5	13	-
t3 increments	-	2048	2048	2048	2048	-	-
sw (t3) / ppm	-	100	100	100	100	-	-
Acq time (t3) / ms	-	12.9	12.9	12.9	12.9	-	-
^{1}H dec. / kHz	10	10	10	10	10	10	5 (WALTZ64)
^{15}N dec. / kHz	10	5	5	5	10	10	-
^{13}C dec. / kHz	10	10	10	10	10	10	-
Water sup. (100 ms) / kHz	15	20	20	20	20	15	-
Interscan delay / s	1.5	1.5	1.5	1.4	1.2	1.5	1.5
Number of scans	80	32-64	32-80	64-80	48-128	80	41984-58408
Experiment time	**11h30**	**2-3 days**	**2-3 days**	**2-3 days**	**3-6 days**	**~12h**	

Supplementary Table 5: Experimental parameters for measurement of site-specific 3D hCANH $R_{1\rho}(^{15}\mathrm{N})$ relaxation-rate constants shown in Figure 1d.

**Sample**	**^{2}H-^{13}C-^{15}N-Cp149 + JNJ-890**	**^{2}H-^{13}C-^{15}N -Cp149**
MAS frequency/ kHz	80	80
Field/ T	20	20
**Transfer I**	HC-CP(DQ)	HC-CP(DQ)
^{1}H field/ kHz	64	61
^{13}C field/ kHz	14	15
Shape	Tangent ^{1}H	Tangent ^{1}H
Carrier / ppm	55	52
Time/ ms	5.25	4.5
**Transfer II**	CN-CP	CN-CP
^{13}C field/ kHz	49	50
^{15}N field/ kHz	30	29
Shape	Tangent ^{13}C	Tangent ^{13}C
Carrier/ ppm	117.5	117.5
Time/ ms	18	17
**Transfer III**	NH-CP	NH-CP
^{1}H field/ kHz	60	59
^{15}N field/ kHz	17	17
Shape	Tangent ^{1}H	Tangent ^{1}H
Carrier/ ppm	4.8	4.8
Time/ ms	2	2.4
**$T_{1\rho}(^{15}\mathrm{N})$ Measurement**	13 kHz Spin-Lock ^{15}N	13 kHz Spin-Lock ^{15}N
Relaxation delays / ms	0.001, 26, 51, 101, 126, 151, 201, 251	0.001, 26, 51, 101, 126, 151, 201, 251
t1 increments	50	50
Sweep width (t1)/ ppm	30	30
Acquisition time (t1)/ ms	3.9	3.9
t2 increments	30	30
Sweep width (t2)/ ppm	40	40
Acquisition time (t2)/ ms	4.4	4.4
t3 increments	2048	2048
Sweep width (t3)/ ppm	47	47
Acquisition time (t3)/ ms	25.8	25.8
^{1}H swfTPPM decoupling/ kHz	10	10
^{15}N WALTZ64 decoupling/ kHz	10	5
^{13}C WALTZ64 decoupling/ kHz	5	5
Water Suppression	MISSISIPPI	MISSISIPPI
^{1}H field / kHz	20	20
Time / ms	120	120
Interscan delay/ s	2.19	2.19
Number of scans	24	24
Measurement time/ h	184	184

Code for relaxation rate determination and error estimation

R1rho_3D

```
clear all
close all

addpath('/home/alsi/Documents/MATLAB')
addpath('/home/supe/matlab')
addpath('/home/alma/INFOS/INFOS_v1.0/')
addpath('~/export_fig')

VDS=[1, 26,51, 101,126,151,201,251]

Iall=zeros(76,length(VDS));

for q=1:length(VDS)
    if q==1
     file=sprintf('2HCp149_%0.0fu_80kHz.txt',VDS(q))
    else
    file=sprintf('2HCp149_%0.0fm_80kHz.txt',VDS(q))
    end
[assign,I]  = textread(file,'%s %f %*f %*f %*f %*f %*f');

Iall(:,q)=I';

end

t=[0.001, 26,51, 101,126,151,201,251]*1e-3;

IntTrace_150=Iall' % Retrieve the intensity for each peak and each trace
t90=t;
[t290, SORidx90]=sort(t90);


%% Perform error analysis using the bootstrapping method
residue=1:length(Iall);
    RES=residue;
    npeaks=numel(RES);
    opt.df=2;
    opt.Nboot=500;
    opt.plot='n';
    fit_150=zeros(1,npeaks);
    erp_150=zeros(1,npeaks);
    erm_150=zeros(1,npeaks);
    A_110=zeros(1,npeaks);
    C_110=zeros(1,npeaks);

    for k=1:npeaks
        k
        temp=ExpFitBoot(t290,IntTrace_150(SORidx90,k)',opt);
        fit_150(k)=1/temp.R;
        A_110(k)=temp.A;
                erm_150(k)=-(temp.R+temp.Rstd)+(temp.R);
        erp_150(k)=(temp.R-temp.Rstd)-(temp.R);
    end

    %%
    dock(2)
clf(2)
for k=1:npeaks
    subplot(10,8,k)
    scatter(t290/1e-3,IntTrace_150(SORidx90,k)/(A_110(k)),'ob',
'markerfacecolor','b')
   hold all
    plot(t290/1e-3,(A_110(k)*exp(-t290/fit_150(k)))/(A_110(k)),'-r','linewidth',1)
    axis([0 t290(end)/1e-3 0 1.1])
end
%%
```

```
figure(21)
clf
errorbar(1./(fit_150),erm_150    ,    erp_150    ,'Color',    'b',    'LineStyle','-
','Marker','^','LineWidth',1.7)
ylim([0 18])
hold on

%%
save('R1rho_3D_hCANH_HBvCp149_80kHz_new','erm_150', 'erp_150','fit_150')
```

R1rho_Barplot

```
clear all
close all

errscale=1;

fit=zeros(2,142);
err=zeros(2,142);

load('R1rho_3D_hCANH_HBvCp149_80kHz')
leR=max(assignno);

for i=1:length(assignno)
fit(1,assignno(i))=1./fit_150(i);
err(1,assignno(i))=erm_150(i);
end

load('R1rho_3D_hCANH_HBvCp149_HAP_80kHz')

%eliminate overlap
fit_150(end)=[];
erm_150(end)=[];

for i=1:length(assignno)-1
fit(2,assignno(i))=1./fit_150(i);
err(2,assignno(i))=erm_150(i);
end

% Bar Plot

ymax=15;

figure(2)
a=fit(:,:);
resi=1:length(a);
hBar=bar(resi, a','BarWidth',1)
b=err(:,:)*errscale;

for k1=1:size(a,1)
    colormap(winter)
    ctr(k1,:)=bsxfun(@plus, hBar(1).XData, [hBar(k1).XOffset]')
    ydt(k1,:)=hBar(k1).YData
end
hold on
errorbar(ctr,ydt, b,'.k', 'linewidth',1)
hold off
xlim([0 max(assignno(end-1))+1])
ylim([0 18])
xticks([1:2:max(assignno(end-1))])
xticklabels([1:2:max(assignno(end-1))])
xtickangle(90)
xlabel('Residue')
ylabel('R_{1\rho} (^{15}N) / Hz')
leg=legend('No HAP','With HAP')
set(gca,'fontsize',16)
set(gca,'TickDir','out')
```

```
resi_save=resi';

R1rho=a';

data_save=zeros(length(a),5);
data_save(:,1)=resi';
data_save(:,2:3)=a';
data_save(:,4:5)=abs(b');

save('dyn_data_HAP_no_HAP_002.txt', 'data_save', '-ASCII');

%%
R1rho=a';

data_save=zeros(length(a),5)*NaN;
data_save(:,1)=resi';
data_save(:,2:3)=a';
data_save(:,4:5)=abs(b');

data_save(data_save==0)=NaN;

data_diff=zeros(length(a),3);

data_diff(:,1)=data_save(:,1);
data_diff(:,2)=data_save(:,3)-data_save(:,2);

data_diff(:,3)= sqrt(data_save(:,5).^2 +data_save(:,4).^2);


clear ctr ydt
figure(3)
a=data_diff(:,2);
resi=1:length(a);
hBar=bar(resi, a','BarWidth',1)
b=data_diff(:,3)*errscale;


data_diff(isnan(data_diff))=0;

for k1=1:size(a,2)
    colormap(winter)
    ctr(k1,:)=bsxfun(@plus, hBar(1).XData, [hBar(k1).XOffset]')
    ydt(k1,:)=hBar(k1).YData
end
hold on
errorbar(ctr,ydt, b,'.k', 'linewidth',1)
hold off
xlim([0 max(assignno(end-1))+1])
xticks([1:2:max(assignno(end-1))])
xticklabels([1:2:max(assignno(end-1))])
xtickangle(90)
xlabel('Residue')
ylabel('R_{1\rho} (^{15}N) / Hz')
leg=legend('No HAP','With HAP')
set(gca,'fontsize',16)
set(gca,'TickDir','out')

save('dyn_data_HAP_no_HAP_differences_003.txt', 'data_diff', '-ASCII');
```

ExpFitBoot:

```
function [ out ] = ExpFitBoot( t,I,opt )
%EXPFITBOOT Performs a mono-exponential fit of data, and calculates the
%error using the bootstrap method of error calculation. Exponential fit
%funtion is as follows, for 1, 2, and 3 degrees of freedom (default is 3).
%
%   I = exp( -R*t )           :   df=1
%   I = A*exp( -R*t )         :   df=2
%   I = A*exp( -R*t ) + C     :   df=3
```

```
%
%   I = 1-exp(-R*t)          :   df=-1
%   I = A*(1-exp(-R*t))      :   df=-2
%   I = C-A*exp(-R*t)        :   df=-3
%
%   Where A, R, and C are the fit parameters (Assume that A>0)
%
%   Bootstrapping is performed by acquiring an initial fit, characterized
%   by I_i = f_i() + e_i, where f_i() is a function of R, A, and C
%   (depending on degrees of freedom). Then, a new data set is generated
%   with I_i = f_i() + e_j, where j is a random variable selected from 1 to
%   n, with n being the total number of samples. This is again fit, and the
%   procedure is repeated N times (# of repetitions set in options). Then,
%   a mean and standard deviation are calculate for the R, A, and C.
%
%   Input includes an options variable, with fields as follows:
%
%   opt.df      : Number of degrees of freedom, from 1-3 (default=3)
%                 if df is set to -1 or -3 this will generate a buildup
%                 curve instead of a decay.
%   opt.Nboot   : Number of boot-strap resamplings (default=100)
%   opt.plot    : 'y' or 'n' turns plotting on and off (default 'y')
%
%   out = ExpFitBoot(t,I,opt)
%
%   A. Smith, May 2014


%% Set default options if not specified.
if exist('opt','var')
    if not(isfield(opt,'df'))
        opt.df=3;
    end
    if not(isfield(opt,'Nboot'))
        opt.Nboot=100;
    end
    if not(isfield(opt,'plot'))
        opt.plot='y';
    end
else
    opt=struct('df',3,'Nboot',100,'plot','y');
end

%% Sort time vector and data
[t,b]=sort(t(1:end));
I=I(b);

%% Setup fit functions and initial guess
switch opt.df
    case 1
        fun=@(X)exp(-X*t);
        [~,b]=min(abs(I-.3679));
        if t(b)==0
            X00=1/t(end)*2;
        else
            X00=1/t(b);
        end
    case 2
        fun=@(X)X(2)*exp(-X(1)*t);
        A0=max(I);
        [~,b]=min(abs(I/A0-.3679));
        if t(b)==0
            X00=[1/t(end)*2 A0];
        else
            X00=[1/t(b) A0];
        end
    case 3
        fun=@(X)X(3)+X(2)*exp(-X(1)*t);
        C0=min(I);
        A0=max(I)-C0;
```

```
        [~,b]=min(abs((I-C0)/A0-.3679));
        if t(b)==0
            X00=[1/t(end)*2 A0 C0];
        else
            X00=[1/t(b) A0 C0];
        end
    case -1
        fun=@(X)1-exp(-X*t);
        [~,b]=min(abs(I-.6321));
        if t(b)==0
            X00=1/t(end)*2;
        else
            X00=1/t(b);
        end
    case -2
        fun=@(X)X(2)*(1-exp(-X(1)*t));
        A0=max(I);
        [~,b]=min(abs(I/A0-.6321));
        if t(b)==0
            X00=[1/t(end)*2 A0];
        else
            X00=[1/t(b) A0];
        end
    case -3
        fun=@(X)X(3)-X(2)*exp(-X(1)*t);
        C0=max(I);
        A0=max(I)-min(I);
        [~,b]=min(abs((C0-I)/A0-.3679));
        if t(b)==0
            X00=[1/t(end)*2 A0 C0];
        else
            X00=[1/t(b) A0 C0];
        end
end

%% Acquire initial fit
opt.Display='off';
minfun=@(X)sum((fun(X)-I).^2);
X0=fminsearch(minfun,X00,opt);
error=fun(X0)-I;

%% Run bootstrap loop
X=zeros(opt.Nboot,abs(opt.df));
np=length(I);

% if matlabpool('size')>0
%     parfor k=1:opt.Nboot
%         rand_ind=randi(np,1,np);
%         minfun=@(X)sum((fun(X0)+error(rand_ind)-fun(X)).^2);
%         X(k,:)=fminsearch(minfun,X0,opt);
%     end
% else
    for k=1:opt.Nboot
        rand_ind=randi(np,1,np);
        minfun=@(X)sum((fun(X0)+error(rand_ind)-fun(X)).^2);
        X(k,:)=fminsearch(minfun,X0,opt);
    end
%end
%% Output results
Xavg=mean(X);
Xstd=std(X);

switch opt.df
    case 1
%           out.R=Xavg;
        out.R=X0(1);
        out.Rstd=Xstd;
        out.rms=sqrt(1/length(t)*sum((exp(-t*out.R)-I).^2));
    case 2
%           out.R=Xavg(1);
```

```
%          out.A=Xavg(2);
        out.R=X0(1);
        out.A=X0(2);
        out.Rstd=Xstd(1);
        out.Astd=Xstd(2);
        out.rms=sqrt(1/length(t)*sum((out.A*exp(-t*out.R)-I).^2));
    case 3
%          out.R=Xavg(1);
%          out.A=Xavg(2);
%          out.C=Xavg(3);
        out.R=X0(1);
        out.A=X0(2);
        out.C=X0(3);
        out.Rstd=Xstd(1);
        out.Astd=Xstd(2);
        out.Cstd=Xstd(3);
        out.rms=sqrt(1/length(t)*sum((out.A*exp(-t*out.R)+out.C-I).^2));
    case -1
        out.R=X0(1);
        out.Rstd=Xstd(1);
        out.rms=sqrt(1/length(t)*sum(((1-exp(-t*out.R))-I).^2));
    case -2
        out.R=X0(1);
        out.A=X0(2);
        out.Rstd=Xstd(1);
        out.Astd=Xstd(2);
        out.rms=sqrt(1/length(t)*sum((out.A*(1-exp(-t*out.R))-I).^2));
    case -3
        out.R=X0(1);
        out.A=X0(2);
        out.C=X0(3);
        out.Rstd=Xstd(1);
        out.Astd=Xstd(2);
        out.Cstd=Xstd(3);
        out.rms=sqrt(1/length(t)*sum(((out.C-out.A*exp(-t*out.R))-I).^2));
end

out.X=X;



%% Plot results
if strcmpi(opt.plot(1),'y')
    if not(isempty(get(gcf,'Children')))
        figure
    end

    scatter(t,I,'MarkerEdgeColor','Red')
    hold all
    plot(t,fun(X0),'Color','Blue')
    axis tight

    if not(exist('C0','var'))
        C0=0;
    end
    if not(exist('A0','var'))
        A0=1;
    end

    switch opt.df
        case 1
            text(t(1)+(t(end)-t(1))*.6,C0+.7*A0,['R=' num2str(out.R,'%3.3g') ' +/- '
num2str(out.Rstd,'%2.2g')])
            title('Fit to I=exp(-R*t)')
        case 2
            text(t(1)+(t(end)-t(1))*.6,C0+.7*A0,['R=' num2str(out.R,'%3.3g') ' +/- '
num2str(out.Rstd,'%2.2g')])
            text(t(1)+(t(end)-t(1))*.6,C0+.55*A0,['A=' num2str(out.A,'%3.3g')  ' +/-
' num2str(out.Astd,'%2.2g')])
            title('Fit to I=A*exp(-R*t)')
```

```
        case 3
            text(t(1)+(t(end)-t(1))*.6,C0+.7*A0,['R=' num2str(out.R,'%3.3g') ' +/- ' num2str(out.Rstd,'%2.2g')])
            text(t(1)+(t(end)-t(1))*.6,C0+.55*A0,['A=' num2str(out.A,'%3.3g') ' +/- ' num2str(out.Astd,'%2.2g')])
            text(t(1)+(t(end)-t(1))*.6,C0+.4*A0,['C=' num2str(out.C,'%3.3g') ' +/- ' num2str(out.Cstd,'%2.2g')])
            title('Fit to I=A*exp(-R*t)+C')
        case -1
            text(t(1)+(t(end)-t(1))*.6,C0+.7*A0,['R=' num2str(out.R,'%3.3g') ' +/- ' num2str(out.Rstd,'%2.2g')])
            title('Fit to I=1-exp(-R*t)')
        case -2
            text(t(1)+(t(end)-t(1))*.6,C0+.7*A0,['R=' num2str(out.R,'%3.3g') ' +/- ' num2str(out.Rstd,'%2.2g')])
            text(t(1)+(t(end)-t(1))*.6,C0+.55*A0,['A=' num2str(out.A,'%3.3g') ' +/- ' num2str(out.Astd,'%2.2g')])
            title('Fit to I=A*(1-exp(-R*t))')
        case -3
            text(t(1)+(t(end)-t(1))*.6,C0-.7*A0,['R=' num2str(out.R,'%3.3g') ' +/- ' num2str(out.Rstd,'%2.2g')])
            text(t(1)+(t(end)-t(1))*.6,C0-.55*A0,['A=' num2str(out.A,'%3.3g') ' +/- ' num2str(out.Astd,'%2.2g')])
            text(t(1)+(t(end)-t(1))*.6,C0-.4*A0,['C=' num2str(out.C,'%3.3g') ' +/- ' num2str(out.Cstd,'%2.2g')])
            title('Fit to I=C-A*exp(-R*t)')
    end
end


end
```

Source files – gels and western blots

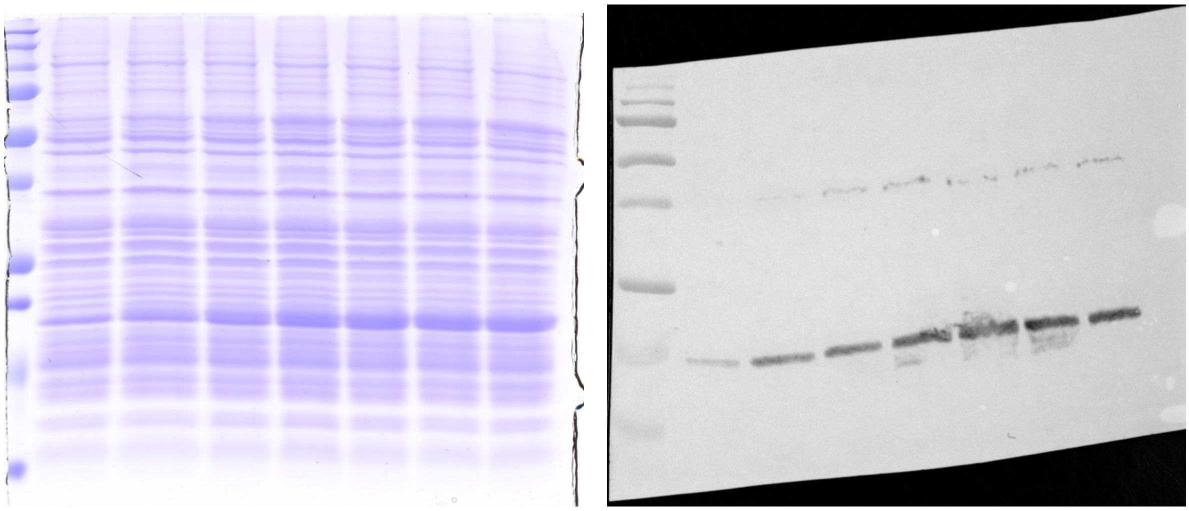

Uncropped SDS-PAGE and Western-Blot shown in Supplementary Figure 13. Antibody= a-c149.

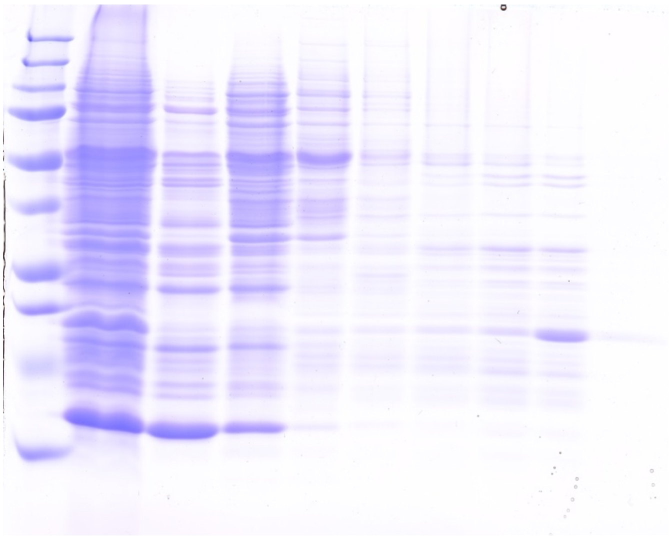

Uncropped SDS-PAGE shown in Supplementary Figure 14a.

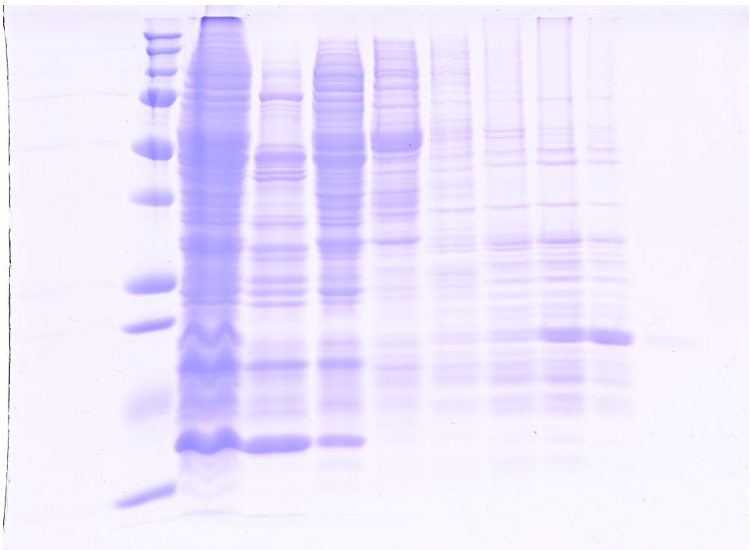

Uncropped SDS-PAGE shown in Supplementary Figure 14b.

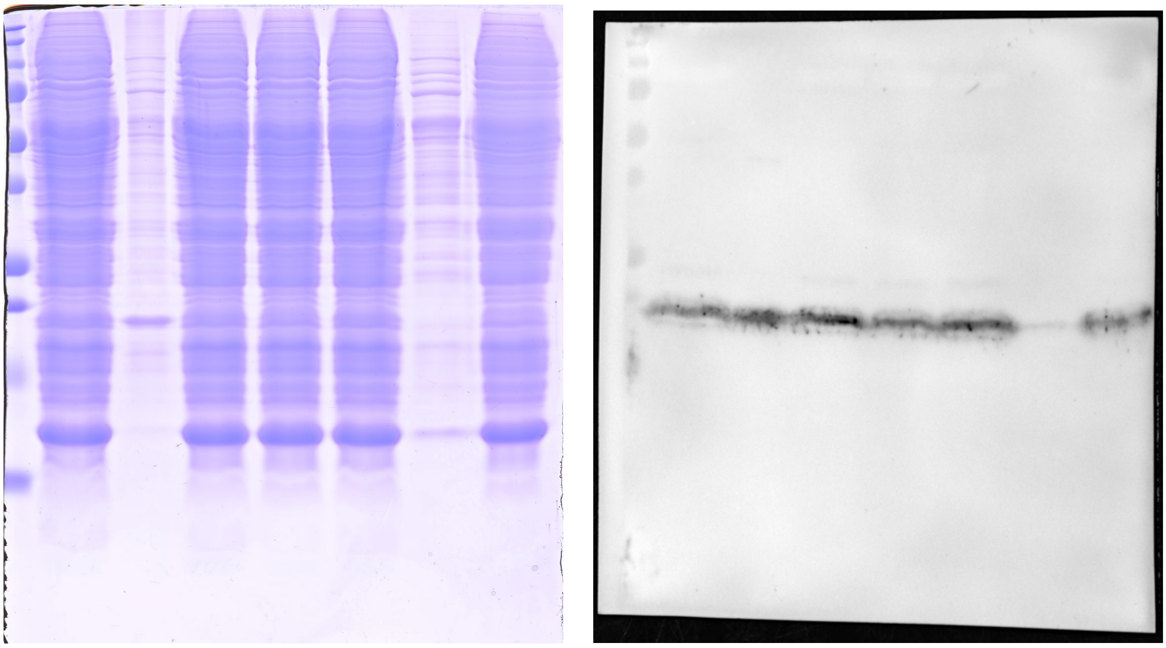

Uncropped SDS-PAGE and Western Blot shown in Supplementary Figure 14c. Antibody= a-c149.

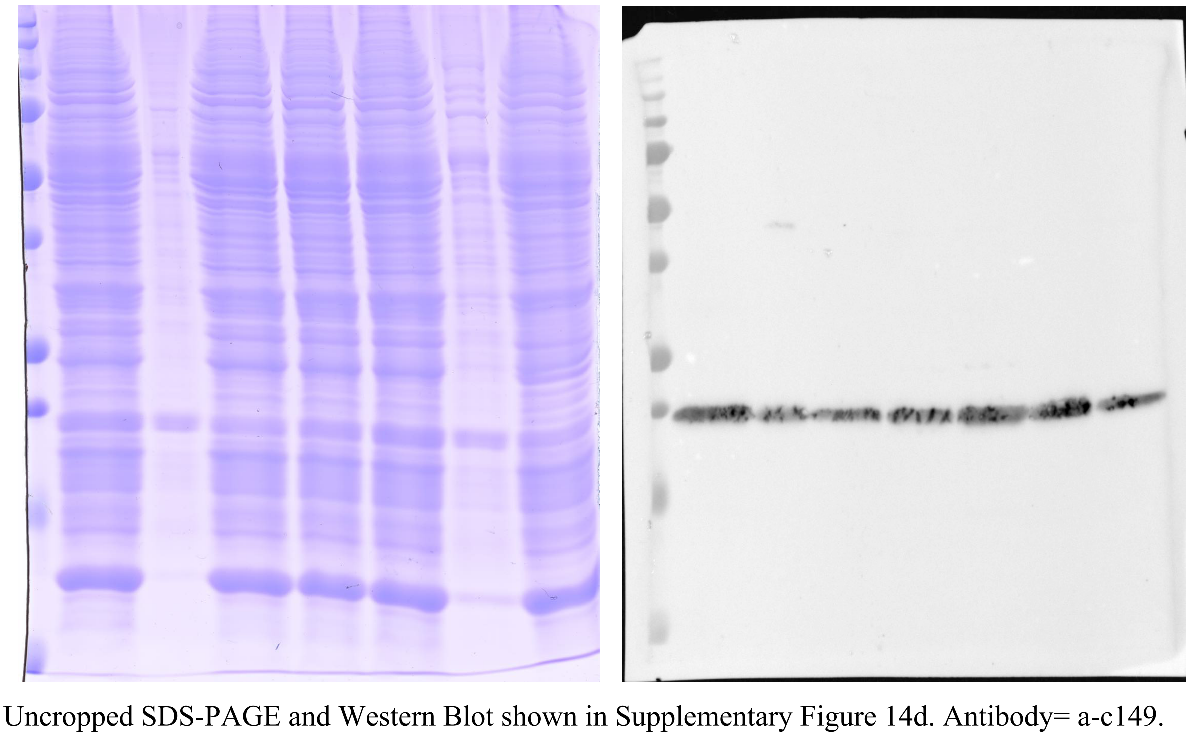

Uncropped SDS-PAGE and Western Blot shown in Supplementary Figure 14d. Antibody= a-c149.

References

1. Lecoq, L. *et al.* A pocket-factor–triggered conformational switch in the hepatitis B virus capsid. *Proc Natl Acad Sci USA* **118**, e2022464118 (2021).
2. Venkatakrishnan, B. *et al.* Hepatitis b virus capsids have diverse structural responses to small-molecule ligands bound to the heteroaryldihydropyrimidine pocket. *Journal of virology* **90**, 3994–4004 (2016).
