# Supplementary material for: Molecular elucidation of drug-induced abnormal assemblies of the hepatitis B virus capsid protein by solid-state NMR
Source: Nat Commun. 2023 Jan 28;14:471. doi: 10.1038/s41467-023-36219-3 (PMC9884277; doi:10.1038/s41467-023-36219-3)
Supplement: Supplementary file 3 — Reporting Summary [file 41467_2023_36219_MOESM3_ESM.pdf]

nature portfolio

Corresponding author(s): Anja Böckmann
Michael Nassal
Beat H Meier

Last updated by author(s): Nov 04 2023

Reporting Summary

Nature Portfolio wishes to improve the reproducibility of the work that we publish. This form provides structure for consistency and transparency in reporting. For further information on Nature Portfolio policies, see our Editorial Policies and the Editorial Policy Checklist.

Statistics

For all statistical analyses, confirm that the following items are present in the figure legend, table legend, main text, or Methods section.

n/a	Confirmed	
☐	☒	The exact sample size (n) for each experimental group/condition, given as a discrete number and unit of measurement
☒	☐	A statement on whether measurements were taken from distinct samples or whether the same sample was measured repeatedly
☒	☐	The statistical test(s) used AND whether they are one- or two-sided<br>*Only common tests should be described solely by name; describe more complex techniques in the Methods section.*
☒	☐	A description of all covariates tested
☒	☐	A description of any assumptions or corrections, such as tests of normality and adjustment for multiple comparisons
☐	☒	A full description of the statistical parameters including central tendency (e.g. means) or other basic estimates (e.g. regression coefficient) AND variation (e.g. standard deviation) or associated estimates of uncertainty (e.g. confidence intervals)
☒	☐	For null hypothesis testing, the test statistic (e.g. F, t, r) with confidence intervals, effect sizes, degrees of freedom and P value noted<br>*Give P values as exact values whenever suitable.*
☒	☐	For Bayesian analysis, information on the choice of priors and Markov chain Monte Carlo settings
☒	☐	For hierarchical and complex designs, identification of the appropriate level for tests and full reporting of outcomes
☒	☐	Estimates of effect sizes (e.g. Cohen's d, Pearson's r), indicating how they were calculated

Our web collection on statistics for biologists contains articles on many of the points above.

Software and code

Policy information about availability of computer code

Data collection	NMR data acquisition: Bruker Topspin available at https://www.bruker.com/service/support-upgrades/software-downloads/nmr/free-topspin-processing/nmr-topspin-license-for-academia.html
Data analysis	NMR data processing: CCpNMR v 2.4, https://ccpn.ac.uk/software/version-2/version-2-downloads/; code for relaxation analysis is given in SI; MATLAB version R2019a.

For manuscripts utilizing custom algorithms or software that are central to the research but not yet described in published literature, software must be made available to editors and reviewers. We strongly encourage code deposition in a community repository (e.g. GitHub). See the Nature Portfolio guidelines for submitting code & software for further information.

Data

Policy information about availability of data

All manuscripts must include a data availability statement. This statement should provide the following information, where applicable:

- Accession codes, unique identifiers, or web links for publicly available datasets
- A description of any restrictions on data availability
- For clinical datasets or third party data, please ensure that the statement adheres to our policy

The CPSs and relaxation source data underlying Fig. 1f and Supplementary Figs. 1b, 3a, 5a, and 15 are provided as Source Data file. Fits for individual relaxation decays are given in the SI. NMR spectra are available from A.B. upon request. Previously published PDB codes referred to in this manuscript are: 1QGT [10.2210/pdb1QGT/pdb]; 5D7Y [10.2210/pdb5D7Y/pdb]; 5WRE, [0.2210/pdb5WRE/pdb].

Human research participants

Policy information about studies involving human research participants and Sex and Gender in Research.

Reporting on sex and gender	No sex and gender issues are present.
Population characteristics	No populations have been studied.
Recruitment	No participants were recruited
Ethics oversight	No ethics issues were involved

Note that full information on the approval of the study protocol must also be provided in the manuscript.

Field-specific reporting

Please select the one below that is the best fit for your research. If you are not sure, read the appropriate sections before making your selection.

☒ Life sciences ☐ Behavioural & social sciences ☐ Ecological, evolutionary & environmental sciences

For a reference copy of the document with all sections, see nature.com/documents/nr-reporting-summary-flat.pdf

Life sciences study design

All studies must disclose on these points even when the disclosure is negative.

Sample size	Protein sample sizes were determined by NMR rotor size, and are on the order of milligrams, i.e. about 1 uM of proteins.<br>For micrographs, several different regions (>3) were obtained from different regions for each sample.
Data exclusions	No data were excluded
Replication	Replication numbers for all experiments are given in the corresponding Figure Captions. NMR spectra are intrinsically reproducible through signal-averaging using a large number of scans, with signal intensities submitted to variations limited by the the accumulated noise.
Randomization	Multidimensional NMR rarely uses n>1, since experiments are very long (here up to 7.7 days). This is a general issue in NMR, where in cases when signal/noise ratios are small, individual acquisition blocks of experiments are scrutinized to see whether each block shows reproducible data (with n up to around 4). But then, data are added afterwards for the sake of signal/noise. Here, mainly single blocks using one full phase cycle were recorded since signal/noise ratios were sufficient, or experiments too long to record other full phase cycles. While it is well known that NMR equipment is highly expensive, it might be less clear that also sample preparation is expensive (several thousands of €). In such situations, carrying out a study a statistically relevant number of times is not possible. This is why an estimate of the sampling distribution can be obtained by reusing the data from the actual study, over and over again. This is called bootstrapping, which has become an accepted way to get reliable estimates of standard errors and confidence intervals; in fact, it is often considered to be the "gold standard" against which various approximation formulas for standard errors and confidence intervals are judged. (Adapted from https://www.dummies.com/article/academics-the-arts/science/biology/the-bootstrap-method-for-standard-errors-and-confidence-intervals-164614/).
Blinding	This study does not include any clinical trial or research. Blinding does not apply here due to the lack of categories, and since no manual counting or scoring was used to obtain the data.

Reporting for specific materials, systems and methods

We require information from authors about some types of materials, experimental systems and methods used in many studies. Here, indicate whether each material, system or method listed is relevant to your study. If you are not sure if a list item applies to your research, read the appropriate section before selecting a response.

Materials & experimental systems

n/a	Involved in the study
☐	☒ Antibodies
☒	☐ Eukaryotic cell lines
☒	☐ Palaeontology and archaeology
☒	☐ Animals and other organisms
☒	☐ Clinical data
☒	☐ Dual use research of concern

Methods

n/a	Involved in the study
☒	☐ ChIP-seq
☒	☐ Flow cytometry
☒	☐ MRI-based neuroimaging

Antibodies

Antibodies used: polyclonal rabbit antiserum against the assembly domain of the HBV core protein (a-c149) was used for detection of Cp183 (Eurogentec, custom antibody).

Validation: N/A
